# Competition for resources can reshape the evolutionary properties of spatial structure

**DOI:** 10.1371/journal.pcbi.1012542

**Published:** 2024-11-22

**Authors:** Anush Devadhasan, Oren Kolodny, Oana Carja

**Affiliations:** 1 Computational Biology Department, School of Computer Science, Carnegie Mellon University, Pittsburgh, Pennsylvania, United States of America; 2 Department of Ecology, Evolution, and Behavior, E. Silberman Institute of Life Sciences, The Hebrew University of Jerusalem, Jerusalem, Israel; Abdus Salam International Centre for Theoretical Physics, ITALY

## Abstract

Many evolving ecosystems have spatial structures that can be conceptualized as networks, with nodes representing individuals or homogeneous subpopulations and links the patterns of spread between them. Prior models of evolution on networks do not take ecological niche differences and eco-evolutionary interplay into account. Here, we combine a resource competition model with evolutionary graph theory to study how heterogeneous topological structure shapes evolutionary dynamics under global frequency-dependent ecological interactions. We find that the addition of ecological competition for resources can produce a reversal of roles between amplifier and suppressor networks for deleterious mutants entering the population. We show that this effect is a nonlinear function of ecological niche overlap and discuss intuition for the observed dynamics using simulations and analytical approximations. We use these theoretical results together with spatial representations from imaging data to show that, for ductal carcinoma, where tumor growth is highly spatially constrained, with cells confined to a tree-like network of ducts, the topological structure can lead to higher rates of deleterious mutant hitchhiking with metabolic driver mutations, compared to tumors characterized by different spatial topologies.

## Author summary

Many evolving ecosystems are characterized by topological constraints and heterogeneous spatial architectures. For example, in ductal carcinoma, tumor growth is highly spatially constrained, with cells confined to a tree-like network of ducts. Here we use the mathematical formalism of networks to study how heterogeneity in population structure shapes rates of evolution, and specifically, probabilities of fixation of new mutants in the population. Using simulations and analytic approximations, together with mathematical representations of architectures inferred from imaging studies, we show that the complex, heterogeneous topology of many real world evolving ecosystems can critically reshape their genetic makeup. In particular, we show that there are topological structures that, under particular ecological conditions, can reshape rates of accumulation of deleterious mutations in a population, compared to systems that are well-mixed or have lattice-based structures of growth. Our work develops an initial framework to understand the effect of the population topology on eco-evolutionary dynamics, as well as helps inform on how to spatially engineer these types of ecosystems to select for evolutionary outcomes of interest.

## Introduction

Coexistence is a hallmark of many natural systems and the maintenance of biodiversity is a central theme in ecology and conservation biology [1, 2]. The mechanisms of coexistence can often be traced to differences in resource utilization that circumvent competitive exclusion and allow longterm stable maintenance of two or more ecotypes in the population [3–7]. Until recently, most models assumed the time scales of ecology and evolution to be non-overlapping, and rarely considered the role of constitutively beneficial or deleterious fitness mutations and stochastic evolutionary dynamics in perturbing these ecological equilibria [8, 9]. However, it has become increasingly clear that rapid evolution can occur on the timescale of ecological niche diversification, for example through the stochastic accumulation of adaptive mutations within ecotype clades, resulting in fixation of particular lineages and the eventual loss of coexistence [10–14].

While it is increasingly theoretically and empirically recognized that ecological interactions can shape evolutionary dynamics and vice versa, studies exploring these eco-evolutionary interactions usually assume well-mixed populations and no spatial heterogeneity [13] or simple spatial structures with high degrees of symmetry and regularity, such as connected demes, lattices or an infinite number of patches that are all connected by uniform dispersal [15]. However, the structure of natural populations is often ‘messy’ and patchy, with empirical work reinforcing the importance of heterogeneous networks of interaction and replacement in shaping the genetic makeup of eco-evolutionary systems [16–19]. For example, in ductal carcinoma, tumor growth is highly spatially constrained by the complex branching tissue architecture of the pancreatic ductal network [20]. Similarly, heterogeneous spatial and temporal gradients have been shown to interact to shape the dynamics of plant–pollinator networks [21] and the complex branching topology of riverine ecosystems has been shown to be critical for biodiversity maintenance [22]. Microbial spread and colonization of the nasal turbinates has been shown to be patchy, with single bacterial cells seeding the surface of epithelia at seemingly random locations [23] and we are just beginning to understand how surface topography, i.e. the network structure of contact between different locations, determines the importance of spatial pattern and scale in shaping evolutionary dynamics in commensal microbial communities within our bodies [24, 25].

Here we use the mathematical formalism of networks to study how heterogeneity in population structure shapes eco-evolutionary dynamics. Each node in the graph represents one individual in the population, or a single well-mixed subpopulation, and edges are proxies for the local pattern of mutant spread. Networks make for a natural and versatile mathematical representation of heterogenous population structure because they allow for continuous tuning of the complexity of the population topology: the limit of a well-mixed population can be represented using the complete graph, other regular structures, such as lattices, can be represented using k-regular graphs and, through repeated and systematic deletion or addition of links between nodes, we can design population topologies with varying degrees of asymmetry and complexity, or mathematically represent novel structures learned from patchy, irregular natural biological architectures.

Another benefit of using the mathematical proxy of networks is the existence of a previous body of theoretical work in evolutionary graph theory (EGT), focused on the study of how graph structure shapes evolutionary dynamics, in the absence of ecological niche diversification and competition for resources. These prior models have shown that the addition of heterogeneity to population structure can greatly extend the range of possible evolutionary outcome beyond what is possible with regular deme and island-based models [26–31]. In previous work, we have shown that the evolutionary properties of a graph can be understood by how they change the rate of evolution of a new mutant appearing on the network, compared to a well-mixed population and, specifically, by analyzing two essential network properties: the network amplification factor, which shapes probabilities of fixation compared to well-mixed populations [29, 31] and the network acceleration factor, which shapes the time to fixation for new mutations in the population [30, 31].

**Fig 1:**
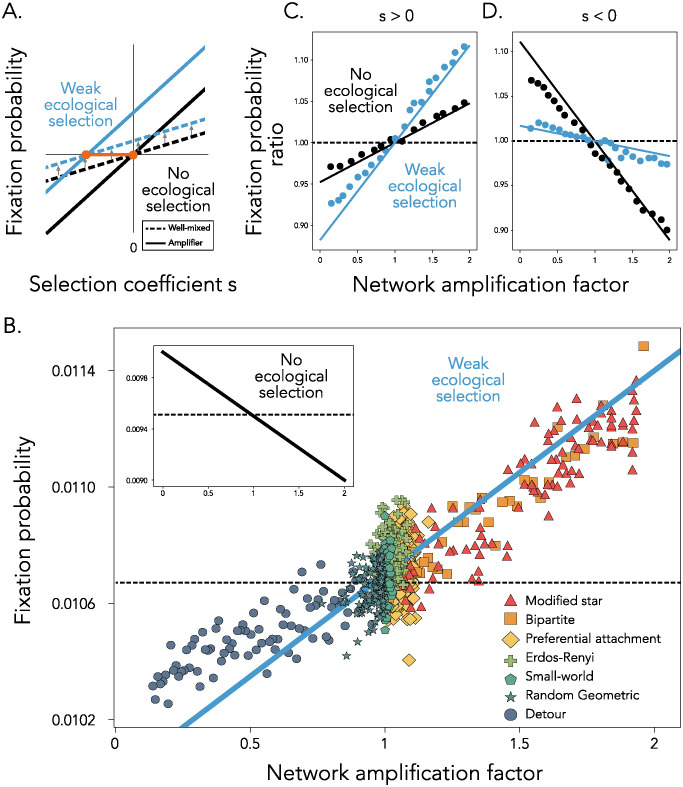
The role of network topology in shaping mutant fixation probabilities for generalist populations. **Panel A** shows an illustration of the effect of weak ecological selection on the probability of fixation. Fixation probability is shown on the y-axis and selection strength on the x-axis. One set of black dashed (well-mixed) and continuous (amplifier) lines show the fixation probability under no ecological selection, while the other set in blue showcases the regime with weak ecological selection. The orange points show the points of intersection for the two ecological regimes. **Panel B** Fixation probability on the y-axis as a function of network amplification factor on the x-axis, across network families. Each dot represents the fixation probability of a single network, calculated using 10^6^ simulation runs. The dashed line represents Eq (6) for a well-mixed population, while the solid blue line shows Eq (7) for graph topologies. Here, *s* = −0.001, *N* = 100 and *α* = 0.53 (blue solid line) and *α* = 0.5 for the insert. In **Panels C** and **D**, dots show the ratio of fixation probabilities across network families and the well-mixed population baseline, as a function of network amplification factor, for a beneficial (*s* = 0.001, **Panel C**) and deleterious (*s* = −0.002, **Panel D**) mutant, estimated using 10^6^ simulation runs. Lines show the analytic approximations using Eqs (6) and (7). Here, *N* = 100 and *α* = 0.525 (blue lines).

For example, for a new beneficial mutation entering the population, the network amplification factor *a*_*Bd*_ quantifies how to rescale its selection coefficient *s* for the well-mixed model, in order to obtain the same probability of fixation as an allele with selection coefficient *s* on the network population. In other words, a new mutation with a selective benefit *s* appearing in a graph population, would have a probability of fixation corresponding to an equivalent selection coefficient *a*_*Bd*_*s* in a well-mixed population. If *a*_*Bd*_ > 1, the graph amplifies selection, if *a*_*Bd*_ < 1 the graph suppresses selection and if *a*_*Bd*_ = 1 the graph does not change probabilities of fixation compared to the well-mixed. Moreover, an amplifier of selection boosts the selective benefit of new beneficial mutants, compared to a well-mixed population, and lowers it if the mutant is deleterious, with the switch occurring precisely at neutrality, *Ns* = 0 [29, 31] (see also Fig 1A). The opposite is true for suppressor topologies [26, 29, 31]. Therefore, evolutionary graph theory classifies population topologies as one of three types: amplifiers, isothermal (a class which includes lattices and other regular structures) or suppressors of selection. At a big picture level, this is because heterogeneity in the number of neighbors each node in the graph has creates heterogeneity in node reproductive advantage, with some nodes more likely to reproduce than others and some more likely to die [29].

Here we discuss how this simple, but powerful classification breaks down in significant and unintuitive ways when the system is also characterized by global frequency-dependent ecological interactions. We develop and investigate a model that combines the EGT framework with a resource competition model and study how the existence of ecological niche differences reshapes probabilities of fixation of new mutants in network-structured populations. In the limiting case of complete niche overlap, our model recovers the constant directional selection case. With decreasing niche overlap however, we find that the interplay between frequency-dependent ecological selection and frequency-independent directional selection makes spatial structures that are amplifiers of selection increase probabilities of fixation for weakly deleterious mutants, in contrast to previous results in EGT. Similarly, we observe that suppressors of selection can now decrease the probabilities of fixation of weakly deleterious mutants.

These results showcase that the topological structure of a fast evolving ecosystem can reshape rates of accumulation of deleterious mutations in a population, compared to well-mixed or lattice-based structures. In addition to the theoretical importance of our results, we also apply our model to show how tissue architecture can significantly impact probabilities of fixation of deleterious mutants hitchhiking on different types of genetic backgrounds in tumor cellular populations. In ductal carcinoma, tumor growth is highly spatially constrained, with cells confined to a tree-like network of ducts [20, 32]. Moreover, metabolic alterations are commonly observed. Specifically, mutations in the KRAS gene may allow cancer cells to substitute glucose with uridine via up-regulation of UPP1 [32, 33]. These mutations can be thought of as metabolic drivers: while prior models usually consider driver mutations to have a constant selective benefit [34, 35], these mutants may initially be strongly beneficial, since they give cells access to a more abundant nutrient source, but may ultimately give rise to frequency-dependent dynamics, due to competition for substitutable resources (glucose and uridine). We use pancreatic ductal networks that have previously been inferred from imaging studies [36] and show that, on these structures, deleterious mutations are more likely to fix when hitchhiking with metabolic drivers, compared to well-mixed or lattice-structured tumor cellular populations.

Our results demonstrate that the complex, heterogeneous topology of many real world evolving ecosystems, ranging from tumor ecosystems, to complex wastewater networks that harbor antibiotic-resistant microbes and riverine ecosystems with intricate branching structures, in interplay with the structure of ecological interaction, can critically reshape their genetic makeup. Our work develops an initial framework to understand the effect of the population topology on eco-evolutionary dynamics, as well as informs on how to spatially engineer these types of ecosystems to select for evolutionary outcomes of interest.

## Model

To study the role of heterogenous population structure in shaping eco-evolutionary dynamics, we use a resource-competition model in which individuals with different metabolic strategies compete for an assortment of externally supplied resources. This resource-based model has the benefit of capturing the key evolutionary and ecological features observed in natural and experimental populations [10, 37], while remaining analytically tractable across a large range of parameters [38]. We consider the simplest nontrivial scenario of two ecotypes evolving in an environment with just two resources. This two-resource case is sufficient to explore the key qualitative differences introduced by ecological interactions, while allowing for analytical tractability. We assume a population of fixed size *N* and analyze the probability of fixation of a single mutant ecotype (mut) invading an initially homogenous wild-type (wt) population.

To represent heterogeneous population structure, we use unweighted and undirected graphs, where each node in the graph represents one individual in the population and edges are proxies for the local pattern of spread, replacement and substitution. A node can also represent a homogeneous subgroup of individuals and the edges can be viewed as migration corridors between them, with the assumption that the timescale of a mutation traveling between nodes is much larger than the time scale of fixation within a node. While the population is assumed to have a network-based structure of reproduction and replacement, the resource availability is spatially uniform: each individual in the population has equal access to the resources, driving the global frequency-dependent dynamics on the whole network. While, as a starting point, our model assumes well-mixed resources, we also present a relaxation of the assumption of spatially homogeneous resources and show that our results hold in the section **Robustness to diffusible resources**.

The two resources are externally supplied at a constant rate *S*. Each ecotype *σ* (mut or wt) is defined by its resource metabolic utilization strategy, *α*_*σ*_ = (*α*_*σ*1_, *α*_*σ*2_). Following [38], we assume *α*_*σi*_ is proportional to the amount of enzyme molecules each individual of type *σ* has allocated to importing and processing resource *i* and has units of amount of enzyme molecules. Let us denote by *c*_*i*_ the nutrient concentration of resource *i* and by *μ*(*c*_*i*_) the rate of consumption of resource i per enzyme molecule [38]. The product *α*_*σi*_*μ*(*c*_*i*_) gives the per individual rate of consumption of resource *i* and is a quantity that can be experimentally measured [39]. The nutrient concentration dynamics of resource *i* follows

$$\begin{array}{c} \frac{dc_{i}}{dt}=S-\left(n_{\text{wt}}\alpha_{\text{wt},\;\text{i}}+n_{\text{mut}}\alpha_{\text{mut},\;\text{i}}\right)\cdot \mu \left(c_{i}\right), \end{array}$$ (1)

where *n*_*σ*_ is the number of individuals of ecotype *σ* in the population at time *t*. We assume a negligible degradation rate and a linear form for *μ*(*c*_*i*_), *μ*(*c*_*i*_) = *c*_*i*_ [38].

Finally, to incorporate evolutionary dynamics, we introduce an intrinsic fitness parameter, orthogonal to the ecological dimension of resource utilization, that can capture phenotypic differences not directly involved in niche diversification [40, 41]. Specifically, intrinsic fitness represents the intrinsic growth rate of the mutant ecotype in the absence of any ecological interactions, for example when grown in monoculture [41, 42]. Alternatively, it could also represent the growth rate in a regime of saturated resources in which the amount of available resources is much larger than the maximum amount of resources the individuals of the population are able to consume. Without loss of generality, we assume a wild-type intrinsic fitness *s*_*wt*_ = 1 and *s*_*mut*_ = (1 + *s*) for the mutant. We refer to *s* = *s*_*mut*_ − 1 as the intrinsic selection coefficient. We refer to a mutant that has *s* > 0 as beneficial and *s* < 0 as deleterious.

To focus the analysis on how a switch in the preferred resource to one that is under-utilized by the wild-type population affects the evolution of the mutant ecotype, we assume that both species have a fixed metabolic budget and *α*_wt_ = (1 − *α*, *α*) and *α*_mut_ = (*α*, 1 − *α*). In other words, the mutant ecotype *mut* switches the preferred resource of the individual, while leaving the relative utilization of the preferred and secondary resources intact (but see Fig A in S1 Text for a relaxation of the assumption of symmetric resource utilization). The parameter *α* represents the amount of enzyme an individual has allocated to importing and processing its preferred resource, but we can also effectively think of *α* simply as the sole proxy of selection arising from niche overlap between the ecotypes. The parameter *α* ranges from a minimum value of 0.5, which corresponds to no ecological selection dynamics (‘generalist’ phenotypes), to a maximum value of 1, corresponding to a regime of completely orthogonal niches, where each ecotype consumes only one of the two resources (‘specialist’ ecotypes). Technically, we note that the full range of *α* is from 0 to 1. However, in Section A in S1 Text, we show that *α* and 1 − *α* give rise to the same relative mutant ecological fitness and therefore we only consider the range 0.5 to 1. Moreover, multiplying the *α*_*σi*_’s by a constant value will leave the relative mutant fitness unchanged.

We can then write the ecotype fitness arising from competition for resources alone as

$$\begin{array}{c} \tilde{r_{\sigma }}=v_{1}\alpha_{\sigma 1}\mu \left(c_{1}^{*}\right)+v_{2}\alpha_{\sigma 2}\mu \left(c_{2}^{*}\right)=\alpha_{\sigma 1}c_{1}^{*}+\alpha_{\sigma 2}c_{2}^{*}, \end{array}$$ (2)

where $c_{i}^{*}$ is the equilibrium concentration of resource *i*, computed by setting $\frac{dc_{i}}{dt}=0$ and solving for *c*_*i*_ (see more in Section A in S1 Text) and *v*_*i*_ is the contribution of resource *i* to overall biomass production and has units of mass per enzyme concentration [38]. Following [38], we assume all resources have equal “value” and set *v*_*i*_ = 1. Therefore $\tilde{r_{\sigma }}$ has units of mass per time. The relative mutant ecological fitness, that relates to niche partitioning, can be written as $r_{mut}=\frac{\tilde{r_{mut}}}{\tilde{r_{wt}}}$. We will refer to this component of fitness that relates to niche partitioning as the “ecological” component of fitness.

Taking into account the two components contributing to an individual’s fitness, the intrinsic component of fitness and the ecological component, we write the overall fitness of ecotype *σ* as

$$\begin{array}{c} \tilde{f_{\sigma }}=s_{\sigma }\tilde{r_{\sigma }}=s_{\sigma }\left(\alpha_{\sigma 1}\mu \left(c_{1}^{*}\right)+\alpha_{\sigma 2}\mu \left(c_{2}^{*}\right)\right). \end{array}$$ (3)

Therefore selection pressures in our model are represented by two parameters that separately control the strengths of two types of fitness: the directional intrinsic axis of fitness and the frequency-dependent ecological axis of fitness. Their combined effect is captured by the overall fitness, which is the key parameter governing the average reproductive rates of the two ecotypes (see also [13, 14]). Although the above multiplicative overall fitness is a postulated form, it follows from prior models. For instance, the net growth rate in the Lotka-Volterra model is given by the product between an intrinsic growth rate and an ecological component which captures competitive ecological interactions [40]. In addition, many theoretical models in population genetics assume that the individual fitness contributions from multiple loci combine multiplicatively [43]. The relative mutant overall fitness can then be written as $f_{mut}=\frac{\tilde{f_{mut}}}{\tilde{f_{wt}}}$.

To model population dynamics we use a stochastic discrete-time Moran model with Birth-death (Bd) updating. The population is initialized with exactly one mutant, placed on a random node of the population network. At each discrete time step, one individual is sampled for reproduction proportional to fitness, according to Eq (3). The offspring then replaces a randomly selected neighbor of the parent node. This is the essential difference that allows us to study the role of local population structure, in comparison to a well-mixed population: in a well-mixed population, a node from the entire network would be randomly selected for death. Ecological fitness differences arising from niche differences between the two ecotypes introduce negative frequency dependence that pushes the lineages towards an interior equilibrium of coexistence, which also depends on the intrinsic fitness difference *s*. This equilibrium is unstable however and, due to genetic drift, the only stable equilibria are the two absorption points, fixation or loss of the mutant allele.

We develop analytic approaches and use Monte-Carlo simulations with at least 10^6^ runs to estimate the mutant’s fixation probability and conditional fixation time. We simulate the system until fixation or loss of the mutant allele. We systematically explore the space of possible network topologies by generating structures from a variety of network families, including k-regular graphs (including lattice type structures), random spatial geometric graphs, preferential attachment, bipartite and small-world graphs (see Materials and methods for a description of the network families used in this study). To further analyze the space of topologies within graph families, we utilize the concept of the dk-distribution [44] and systematically tune different network properties, independently of one another, across levels of organization. Specifically, we use simulated annealing with edge-swap operations to sample from the full range of a network’s dk-distribution, while keeping all lower levels constant [29].

The list of all model parameters is presented in Table 1.

**Table 1:** List and description of all model parameters.

Parameter	Description
*N*	Population size
*a*_*Bd*_	Property of the network population structure: amplification factor
*c*_*i*_	the nutrient concentration of resource *i*
*μ*(*c*_*i*_)	the rate of consumption of resource i per enzyme molecule
*s*	Intrinsic selection coefficient
*α*	Amount of molecules allocated to importing and processing
	the individual’s preferred resource, niche overlap
$\tilde{r_{\sigma }}$	Ecological fitness of ecotype *σ*
$\tilde{f_{\sigma }}$	Overall fitness of ecotype *σ*
*r*_*mut*_	Relative mutant ecological fitness, $\frac{\tilde{r_{mut}}}{\tilde{r_{wt}}}$
*f*_*mut*_	Relative mutant overall fitness, $\frac{\tilde{f_{mut}}}{\tilde{f_{wt}}}$

## Results

In what follows, we analyze how the network structure changes the mutant’s fixation probability and fixation time, compared to a well-mixed population, as it now operates on two fitness-affecting axes: the constant intrinsic selection coefficient, *s* and the ecological niche overlap, *α*, which gives rise to frequency-dependent selection. We start by describing the eco-evolutionary dynamics in the limit of weak ecological selection, with generalist ecotypes that utilize both resources almost equally and show that the mutant dynamics on the network are shaped by a combined effective selection coefficient that captures the interplay of the two fitness coefficients. We then show that, at the opposite limit of strong ecological selection, with specialist species that have little niche overlap, mutant spread and fixation are shaped by a tradeoff between establishment and conditional fixation probabilities, with the two fitness coefficients playing sequential roles. We then discuss how these two edge regimes provide intuition on the shape of the dynamics observed for ecological interactions of any strength and show that the addition of global frequency-dependent ecological selection pressure can reverse the roles of network topologies traditionally assumed to amplify (and conversely, the ones assumed to suppress) selection.

### The regime of weak ecological selection

In the regime of weak ecological selection, where the variants are generalist species that rely on both available resources almost equally, we show that the interplay between the intrinsic selection coefficient *s* and niche overlap *α* gives rise to a combined effective selective coefficient for the mutant, which we write out below. The network topology further amplifies or suppresses this translated effective selection coefficient (Fig 1A). This can give rise to parameter regimes where the ecological selection makes amplifier network topologies amplify deleterious mutants and suppressor topologies suppress deleterious mutants, relative to a well-mixed population, in contrast to results observed without ecological selection (*α* = 0.5). We recall that a deleterious mutant is one which has a negative intrinsic selection coefficient, i.e. *s* < 0.

Here, weak ecological selection formally refers to the regime $N\cdot \frac{2}{3}{\left(2\alpha -1\right)}^{2}<<1$, with this definition clarified below. In this regime, the global frequency-dependent ecological component of mutant fitness pushes the mutant frequency towards an internal equilibrium, while the constant intrinsic fitness component biases this equilibrium in favor of the fitter ecotype. The mutant frequency at equilibrium is given by

$$\begin{array}{c} x^{*}=\frac{1}{2}+\frac{1}{2}\cdot {\left(2\alpha -1\right)}^{-2}\cdot \frac{s}{\left(2+s\right)}, \end{array}$$ (4)

for *α* > 0.5 (derivation in Section A in S1 Text). When *α* = 0.5, there is no equilibrium because there is no ecological selection. When *s* = 0, the equilibrium frequency is 0.5 for all *α* because neither variant has a fitness advantage. The mutant is more abundant at equilibrium (x* > 0.5) if *s* > 0 and the wild-type is more abundant (x* < 0.5) if *s* < 0.

In the limit of weak ecological and intrinsic selection (|*Ns*| << 1), strong genetic drift makes this equilibrium unstable and the dynamics appear neutral-like. The interplay between the intrinsic fitness coefficient and ecological selection gives rise to a combined effective selective coefficient that is approximately

$$\begin{array}{c} s\left(x\right)\approx s+2{\left(2\alpha -1\right)}^{2}\left(1-2x\right), \end{array}$$ (5)

where x is the mutant frequency in the population (see Section B in S1 Text for this derivation). Therefore, in a well-mixed population, the fixation probability of the mutant becomes approximately

$$\begin{array}{c} \begin{array}{c} P_{fix}^{WM}\approx \frac{1}{N}+\frac{1}{2}s+\frac{1}{3}{\left(2\alpha -1\right)}^{2}. \end{array} \end{array}$$ (6)

The original population under frequency-dependent selection behaves effectively as one under constant selection, with $s_{e}=s+\frac{2}{3}{\left(2\alpha -1\right)}^{2}$. This approximation holds when $|Ns_{e}|=|Ns+N\cdot \frac{2}{3}{\left(2\alpha -1\right)}^{2}|<<1$, [29].

Since the network structure reshapes the mutant’s selection coefficient by increasing or diminishing it proportional to the amplification parameter of the graph, *a*_*Bd*_, the mutant’s new effective selection coefficient becomes *a*_*Bd*_*s*_*e*_. For a spatial structure G, the fixation probability of the new mutant can therefore be approximated as

$$\begin{array}{c} P_{fix}^{G}\approx \frac{1}{N}+\frac{1}{2}a_{Bd}\left(G\right)\left(s+\frac{2}{3}{\left(2\alpha -1\right)}^{2}\right). \end{array}$$ (7)

Fig 1B shows this analytic approximation of the fixation probability (blue line) as a function of *a*_*Bd*_(*G*) on the x-axis, together with simulation results, for a variety of graph families, both suppressors and amplifiers of selection. As a comparison, the insert shows results for the same deleterious mutant with *s* = −0.001, in the absence of ecological selection (*α* = 0.5). When *s*_*e*_ is net positive despite a negative intrinsic selection *s*, i.e. the beneficial contribution from ecological selection is greater than the deleterious contribution from intrinsic selection *s*, an amplifier will promote this deleterious mutant, compared to a well-mixed population. Concretely, this happens when $-\frac{2}{3}{\left(2\alpha -1\right)}^{2}<s<0$. Outside of this region, the effect of the graph topology does not change with the addition of ecological interactions, however the strength of amplification and suppression does change. For beneficial mutants, competition for resources increases the strength of both amplification and suppression (Fig 1C), while the opposite is true for deleterious mutants (Fig 1D).

Note that the effective selection experienced by the mutant is equal to the selection it experiences when it has frequency 1/3 in the population, i.e. $s_{e}=s\left(x=\frac{1}{3}\right)$, as previously observed in other models [45, 46]. We can utilize this result to further generalize the weak selection approximation to cases with unequal resource consumption rates and/or asymmetric resource supply rates (Fig A in S1 Text).

From this result, it also follows that graphs where each node has the same number of neighbors (lattice structures, or more generally, k-regular graphs) obey the isothermal property, since their amplification factor is equal to 1, and they do not change probabilities of fixation compared to a well-mixed topology (Fig B in S1 Text). The isothermal theorem does not usually hold for k-regular graphs in prior models of game-theoretic frequency-dependent dynamics [27]. This is due to the fact that in these prior models frequency dependence is local and the fitness of an individual depends on the frequency of its own type amongst its neighbors [28].

### The regime of strong ecological selection

**Fig 2:**
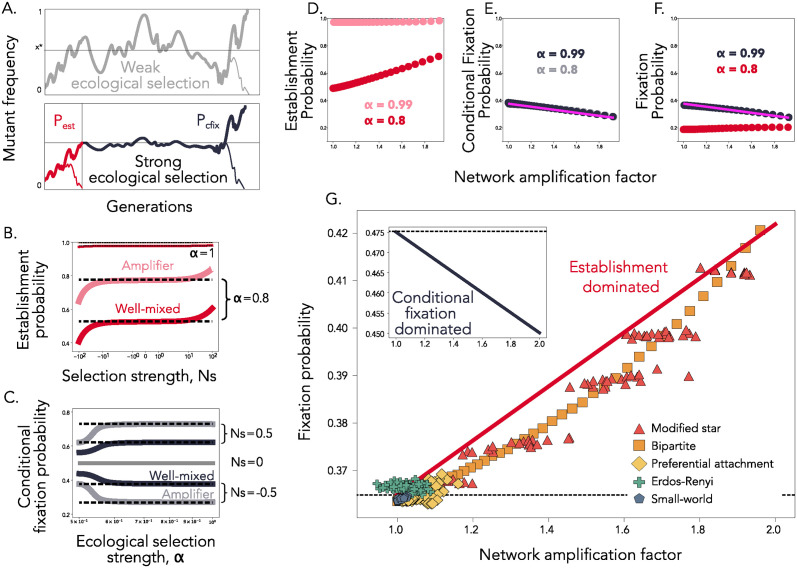
The role of spatial structure in specialist populations. **Panel A** shows toy representations of the mutant trajectory in the generalist (grey) and in the specialist (red/black) regimes. **Panel B** shows the theoretically exact establishment probabilities for well-mixed (solid lines, darker red) and an amplifier (solid lines, lighter red) population (equations (A33) and (A38)). Here, *a*_*Bd*_ = 2, *N* = 500 and *α* = 0.8. The slight change in the establishment probability is also showcased for *α* = 0.99. The corresponding analytic approximations ($P_{est}^{WM}$ and $P_{est}^{S}$) are shown using black dotted lines. **Panel C** shows the theoretically exact conditional fixation probabilities (equations (A32) and (A37)) for a well-mixed (solid lines, darker color) and an amplifier (solid lines, lighter color) population, with amplification factor 2. Here, *N* = 500 and three different values of *Ns*, as depicted in the figure. The corresponding analytic approximations (Eqs (12) and (13)) are shown using black dotted lines. **Panel D** shows establishment probabilities, **Panel E** shows conditional fixation probabilities, and **Panel F** shows total fixation probabilities, as a function of amplification factor, for various PA star networks, computed numerically for two different values of *α*, as shown, and *s* = −0.01. The solid pink line shows the analytic approximation for the conditional fixation probability given by Eq (13). **Panel G** Dots show an approximation of fixation probability using the approach described in the main text on a diverse array of amplifier topologies with varying amplification factors. Here, *N* = 100, *s* = −0.001 and *α* = 0.9. The solid red line shows Eq (14) and the dotted line showcases the well-mixed result as comparison. The insert shows the analytic approximation of the fixation probability, given by Eq (13), for *s* = −0.001 and *α* = 1 (solid black line). The dotted line again shows the well-mixed fixation probability.

In the regime of strong ecological selection both variants are specialist species that consume different resources, with only some niche overlap. The strong frequency-dependent ecological selection makes the mutant persist at the equilibrium frequency for much longer, before eventual fixation or loss from the population (Fig 2A). It is convenient to study the fixation process as the outcome of two independent processes: the probability of the initial establishment of the mutant in the population, followed by the probability of conditional fixation, conditional on mutant frequency starting from the equilibrium frequency [47]. We define establishment as the change from one mutant to Nx* mutants (internal equilibrium) and conditional fixation as the change in mutant frequency from Nx* mutants to N mutants (fixation) (Fig 2A). In what follows, we discuss how network topology, in interplay with the degree of niche differentiation, shapes the time the population spends at this interior equilibrium and how that affects fixation probabilities in this regime. We show that strikingly, the establishment probability is approximately independent of s, and the conditional fixation probability of *α*, for sufficiently large *α*. These approximations hold when $\frac{{\left(2\alpha -1\right)}^{-2}-1+\frac{2}{N}}{{\left(2\alpha -1\right)}^{-2}+1-\frac{2}{N}}<<1$ (which we refer to as the strong ecological selection regime, see Section E in S1 Text and Section F in S1 Text).

In a well-mixed population, the fixation probability is the product of the probabilities of success of both processes: *P*_*fix*_ = *P*_*est*_*P*_*cfix*_. In spatially-structured populations, however, we have to account for all the different ways to arrange Nx* mutants on the graph, at equilibrium. Let **A** be a N x N* matrix in which element (*i*, *j*) is the probability of establishing in the *j* graph equilibrium configuration, given a mutant initialized in node *i*. Here, N* is the total number of spatial equilibrium configurations, ${\text{N}}^{*}=(\begin{array}{c} N\\ Nx^{*} \end{array})$. Let **B** be the vector of size N*, with element *i* equal to the probability of fixation, conditional on establishment in the *i* equilibrium configuration. The fixation probability vector, where element *i* is the fixation probability given mutant initialization in node *i*, is then given by **A** ⋅ **B**. The establishment probability for a graph *G*, i. e. the probability of the mutant establishing in any of the N* equilibrium configurations given that it is initialized on a randomly selected node, becomes

$$\begin{array}{c} P_{est}^{G}=\frac{1}{N}\sum_{i}\sum_{j}\;A_{ij}. \end{array}$$ (8)

Similarly, the conditional fixation probability on a graph can be written as

$$\begin{array}{c} P_{cfix}^{G}=\frac{\sum_{i}\;A_{ij}}{\sum_{i}\sum_{j}\;A_{ij}}\cdot B. \end{array}$$ (9)

The $\frac{\sum_{i}\;A_{\text{ij}}}{\sum_{i}\sum_{j}\;A_{\text{ij}}}$ term is a N*-element vector in which the *i*-th element is the probability of establishing in equilibrium configuration *i*, given that establishment is successful and is a weighted average of the conditional fixation probabilities corresponding to each equilibrium state, where the weights are the relative probabilities of establishment in each of those states. Therefore, for a graph *G*, similar to the well-mixed case, we find that the mutant probability of fixation is the product of the probabilities of success of both establishment and conditional fixation,

$$\begin{array}{c} \begin{array}{cc} P_{est}^{G}\cdot P_{cfix}^{G} & =\frac{1}{N}\sum_{i}\sum_{j}\;A_{\text{ij}}\cdot \frac{\sum_{i}\;A_{\text{ij}}}{\sum_{i}\;\sum_{j}\;A_{\text{ij}}}\cdot B\\ & =\frac{1}{N}\cdot \sum_{i}\;A_{\text{ij}}\cdot B\\ & =\frac{1}{N}\sum_{i}{\left(A\cdot B\right)}_{i}\\ & =P_{fix}^{G}. \end{array} \end{array}$$ (10)

It is therefore sufficient to study these two probabilities independently. In what follows, we discuss how to derive intuitive approximations and also exactly compute these probabilities using exact equations for special case spatial structures (see more in Section D in S1 Text), and methods for probabilities of Markov Chain absorption for a broader class of spatial structures [48, 49].

#### The probability of establishment

For a well-mixed topology, in the limit of weak intrinsic selection coefficient *s* and large population size *N*, the establishment probability can be approximated as

$$\begin{array}{c} P_{est}^{WM}\approx 1-\frac{{\left(2\alpha -1\right)}^{-2}-1+\frac{2}{N}}{{\left(2\alpha -1\right)}^{-2}+1-\frac{2}{N}}, \end{array}$$ (11)

(see derivation in Section E in S1 Text). This approximation is useful, because it shows that *P*_*est*_ does not depend on the intrinsic fitness coefficient, *s* (Fig 2B). Alternatively, we can write $P_{est}^{WM}\approx 1-r_{inv}{\left(\alpha \right)}^{-1}$, where *r*_*inv*_(*α*) is the ecological fitness of the mutant when it has frequency 1/N in the population and note that $P_{est}^{WM}$ is equivalent to the fixation probability of a mutant with relative fitness *r*_*inv*_(*α*). This makes intuitive sense, since *r*_*inv*_(*α*) is the approximate fitness of the mutant in the initial stochastic regime, where genetic drift dominates and before the invasion fitness is significantly impacted by the frequency of the mutant in the population [13]. As in the weak ecological selection regime, establishment can be associated with an effective constant fitness.

A network population structure reshapes this effective fitness *r*_*inv*_(*α*). However, in contrast to the previous regime where the ecological selection pressure is weak, in this case it is harder to obtain general results, except for highly-symmetric topologies. For example, star-structured amplifiers reshape fitness *r* → *r*^2^ [26] and we can therefore approximate the establishment probability on the star as $P_{est}^{S}\approx 1-r_{inv}{\left(\alpha \right)}^{-2}$ (see also Fig C in S1 Text). Note that as *α* approaches 1, ecological selection becomes so strong that establishment occurs almost deterministically, and therefore *P*_*est*_ saturates at 1 for both the well-mixed and star-structured populations (Fig 2B).

#### The probability of conditional fixation

The conditional fixation probability is, in contrast, dominated by the intrinsic selection coefficient *s* and we can write

$$\begin{array}{c} P_{cfix}^{WM}\approx \frac{1-{\left(1+s\right)}^{-N}}{1-{\left(1+s\right)}^{-2N}} \end{array}$$ (12)

(refer to Section F in S1 Text for this derivation). During conditional fixation, spatial structure therefore acts on the intrinsic selection coefficient *s* (Fig 2C) and, for a star-structured population, we can write in closed form $P_{cfix}^{S}\approx \frac{1-{\left(1+2s\right)}^{-N}}{1-{\left(1+2s\right)}^{-2N}}$ (see also Fig C in S1 Text). Note that the star topology boosts the conditional fixation of beneficial mutants and suppresses the fixation of deleterious mutants, as in the constant selection case.

Because conditional fixation is independent of the niche overlap, more general derivations are also possible because we can use the network amplification parameter *a*_*Bd*_ as it reshapes only *s*. For a graph *G* with amplification factor *a*_*Bd*_(*G*), the probability of conditional fixation can be written as

$$\begin{array}{c} P_{cfix}^{G}\approx \frac{1-{\left(1+a_{Bd}\left(G\right)\cdot s\right)}^{-N}}{1-{\left(1+a_{Bd}\left(G\right)\cdot s\right)}^{-2N}}. \end{array}$$ (13)

#### The total probability of mutant fixation

While the frequency dependent dynamics makes the probability of establishment more difficult to analytically compute for topological structures of arbitrary complexity, similar to the case of local frequency dependent dynamics [50, 51], in what follows we show how we can use a combination of simulations and analytic approximations to calculate mutant fixation probability for arbitrarily heterogenous topologies.

For models without global frequency dependence, the probabilities of mutant fixation linearly increase (for *s* > 0) and decrease (for *s* < 0) as a function of the network amplification factor (see insert in Fig 1B for an example *s* < 0 case). In our model, this result holds for advantageous mutants with *s* > 0 because the effect of the topology is the same for both establishment and conditional fixation, i.e. if a spatial structure increases the establishment probability of the mutant, it will also increase its conditional fixation probability, and vice versa. However, here we show that this result does not hold for weakly deleterious mutants with *s* < 0 and the relationship between amplification and mutant fixation probability is fundamentally determined and reshaped by the degree of niche overlap, *α*.

We first study a family of graphs, preferential attachment (PA)-star graphs, for which establishment, conditional fixation, and fixation probabilities can be computed exactly using numerical methods, due to their degree of symmetry [49, 52] (see Materials and methods). Even for the case of a deleterious mutant *s* < 0, the probability of establishment increases with the network amplification factor because the effective constant fitness associated with establishment is beneficial (*r*_*inv*_(*α*) > 1) (Fig 2D). As the population becomes increasingly specialist (*α* → 1), the establishment probability saturates at 1 for all amplification factors, i.e. spatial structure has a negligible effect on establishment in this regime. Separately, the conditional fixation probability decreases with the amplification factor because the effective constant selection coefficient associated with conditional fixation (*a*_*Bd*_*s*) is negative (Fig 2E).

Therefore, the overall fixation probability increases with network amplification factor only in an “establishment dominated regime” (red line, *α* = 0.8 in Fig 2F), where the amplification property of the graph topology increases establishment by a larger amount than it decreases conditional fixation. In contrast, in a “conditional fixation dominated regime”, amplification has almost no effect on establishment, but still decreases conditional fixation by the same amount. Therefore, in this regime, mutant fixation probability is approximately equivalent to the conditional fixation probability, $P_{fix}^{G}\approx P_{cfix}^{G}$ and it is decreased for networks that are traditionally classified as ‘strong’ amplifiers (black line with simulation results and pink numerical approximation results for *α* = 0.99 in Fig 2F).

We can analyze the probability of fixation for a broader class of topologies by computing the establishment and conditional fixation through a combination of simulations and analytics: since the mutant is under strong ecological selection until the equilibrium is reached, the establishment is fast and can be estimated through simulations for more heterogeneous topologies. Separately, Eq (13) provides an approximation for the conditional fixation. In Fig 2G we show that, in the establishment-dominated regime (*α* = 0.9), the fixation probability of the mutant increases with the network amplification factor across a wide array of network families. In contrast, in the regime where the probability of conditional fixation dominates (*α* = 1), network amplification factor decreases the fixation probability (inset Fig 2G for the same intrinsic selection coefficient *s* = −0.001).

In this case, the relationship between the fixation probability and the amplification factor of the network is approximately linear. Since the exact fixation probability can be computed analytically for the well-mixed and star populations, we can use these two points to write a linear approximation for the fixation probability for a spatial structure of arbitrary amplification factor (red line in Fig 2G)

$$\begin{array}{c} P_{fix}^{G}\approx \frac{P_{fix}^{S}}{P_{fix}^{WM}}\left(a_{Bd}-1\right)+P_{fix}^{WM}. \end{array}$$ (14)

**Fig 3:**
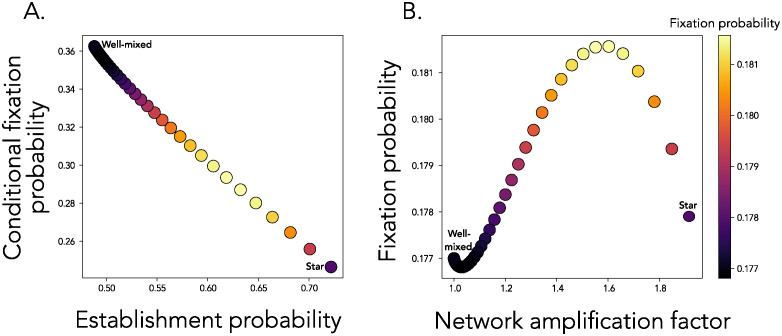
There exist parameter regimes with establishment—Conditional fixation tradeoffs. **Panel A**: Each point shows the conditional fixation probability as a function of the establishment probability for amplifier networks with amplification factor as on the x-axis in Panel B. The total fixation probability is shown by the color of the point, as in the colorbar. In **Panel B**, each point shows the total fixation probability as a function of the amplification factor of the network. For both panels, *α* = 0.8 and *s* = −0.012.

Because fixation is associated with two effective selection coefficients, one for establishment and one for conditional fixation, there also exist parameter regimes where the larger the probability of establishment, the smaller the probability of conditional fixation, and vice versa (Fig 3A), leading to a establishment-conditional fixation tradeoff. A consequence of this tradeoff is that, for weakly deleterious mutants, a nonlinear relationship between the amplification factor of the network and the mutant fixation probability can emerge and fixation probabilities can be maximized by spatial structures with intermediate amplification factors (Fig 3B).

### The strength of resource niche competition determines whether a spatial topology amplifies or suppresses weakly deleterious mutants

**Fig 4:**
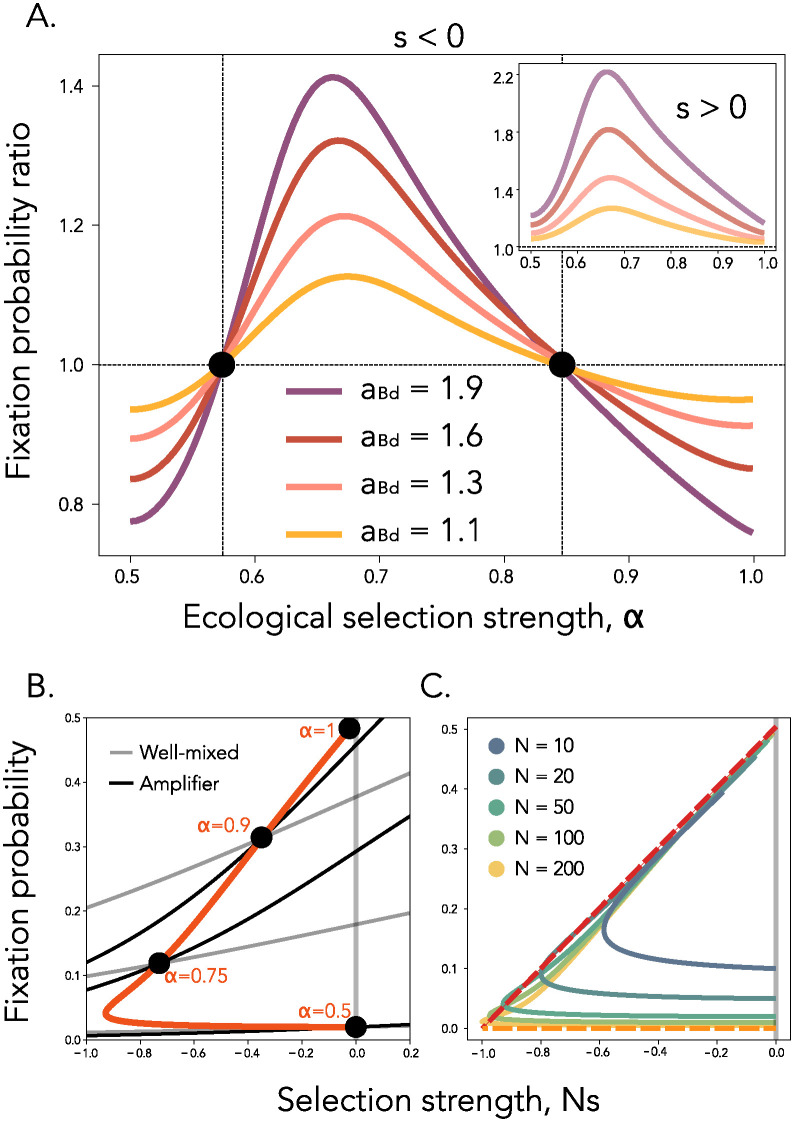
Ecological interactions can reverse the role of amplifiers and suppressors for weakly deleterious mutants. **Panel A.** Ratio of fixation probabilities between various amplifier networks and a well-mixed population. Here, *N* = 50 and *s* = −0.01. The insert shows a beneficial mutant with *s* = 0.01. **Panel B.** Comparison of probabilities of fixation as a function of mutant selection strength for a well-mixed population (grey lines) and an amplifier of selection (black lines, amplification factor equal to 1.92), for four different values of ecological strength *α*, as written in the figure. The orange line follows the exact point of intersection between the well-mixed and amplifier populations, as ecological strength *α* is continuously varied. For *α* = 0.5, we recapture previous results from evolutionary graph theory, where the intersection point is at *Ns* = 0. Here, fixation probabilities are computed exactly, as described in Section D in S1 Text. Population size *N* = 50. **Panel C.** The points of intersection of the fixation probability between well mixed and a star population, for varying population sizes *N*, as depicted in the legend. The orange dotted line shows the approximation for $P_{fix}^{S*}$ in the generalist regime (Section C in S1 Text) and the red dotted line shows the approximation for $P_{fix}^{S*}$ in the specialist regime (see Section G in S1 Text).

A key finding resulting from the analyses above is that topologies that are known amplifiers of selection can also amplify deleterious mutants (*s* < 0) and the strength of ecological selection determines the range of parameters where this result holds (Fig 4A). This unintuitive, nonlinear behavior is in stark contrast to the behavior observed under constant selection models, where amplifier networks decrease the fixation probability of deleterious mutants.

Starting in the limit of *α* = 1, when the two ecotypes have no niche overlap, the initially rare mutant will experience strong positive ecological selection, because it consumes a resource that is completely unused by the wild-type population. Therefore, establishment occurs almost deterministically, regardless of the spatial structure of the population, i.e. $P_{est}^{G}\approx 1$, and the overall probability of fixation is determined by the conditional probability of fixation, $P_{fix}^{G}\approx P_{cfix}^{G}$ and this represents a conditional fixation dominated regime. As *α* decreases, establishment is no longer deterministic and spatial structure will start contributing to the probability of establishment. If the amount by which the graph topology boosts mutant establishment outweighs the amount by which it suppresses conditional fixation, we enter the establishment dominated regime, and the mutant fixation probability on the graph becomes larger than that on a well-mixed structure (second crossing point in Fig 4A). As *α* keeps decreasing, we enter the generalist regime in which, for strong enough ecological differences, the effective selection coefficient $s_{e}=s+\frac{2}{3}{\left(2\alpha -1\right)}^{2}$ is still positive, so the amplifier topology keeps amplifying the deleterious mutant. In the limit of no ecological selection, as *α* approaches 0.5, the effective selection coefficient once more becomes net deleterious and the spatial structure again suppresses the new mutant in the population (first crossing point in Fig 4A). In contrast, amplifiers always increase the fixation probability of beneficial ecotype mutants, in line with previous theory under constant selection (*s* > 0 insert Fig 4A).

Another way to understand this result is through the lens of how *α* determines the intersection point between the fixation probability on a graph topology and on a well-mixed topology as a function of the strength of selection *Ns*. Evolutionary graph theory with constant selection defines amplifiers as topologies that increase the fixation probability of beneficial mutants and decrease that of deleterious ones. Therefore the respective probabilities of fixation intersect at *Ns* = 0 (see Fig 1A black lines). With the addition of global frequency-dependent ecological interactions, this intersection point is shifted to negative values of *Ns*. Specifically, there exists a region of selection against weakly deleterious mutants where ecological selection reverses the evolutionary effect of the spatial structure and amplifiers can favor weakly deleterious mutations and boost their spread compared to well-mixed populations. The extent of this region is maximized for an intermediate value of *α*, such that amplifiers disfavor all deleterious mutations either in the absence of niche differentiation, for *α* = 0.5, or for complete lack of niche overlap and very strong ecological selection, *α* = 1 (Fig 4B). We find that strong amplifiers like the star graph can increase the fixation probability, relative to well-mixed, of deleterious mutants with up to *Ns* ≈ −1 even for small population sizes (Fig 4C). We also show results for suppressor topologies in Fig D in S1 Text.

### Diffusible resources

To relax the assumption that resources are well-mixed, we assume that, at a node *j*, resource *i* is produced at a constant rate *S* and depleted at a rate *α*_*ij*_, where *α*_*ij*_ = *α*_*wt*,*i*_, if node *j* is occupied by a wild-type individual and *α*_*ij*_ = *α*_*mut*,*i*_ otherwise. In addition, we assume resources can diffuse along the edges of the population network with diffusion constant *D*. We can then rewrite Eq (1) as

$$\begin{array}{c} \frac{dc_{ij}}{dt}=S-\alpha_{ij}c_{ij}+D\sum_{k\in nn(j)}(c_{ik}-c_{ij}) \end{array}$$ (15)

where nn(j) is the set of nodes that neighbor node *j* [53]. At equilibrium, when $\frac{dc_{ij}}{dt}=0$, the steady state resource concentrations $c_{ij}^{*}$ obey

$$\begin{array}{c} S=(\alpha_{ij}+k_{j}D)c_{ij}^{*}-D\sum_{k\in nn(j)}c_{ik}^{*}. \end{array}$$ (16)

We can further express the equilibrium concentrations in matrix form as

$$\begin{array}{c} S_{i}=(DL+\text{diag}(\alpha_{i}))c_{i}^{*}. \end{array}$$ (17)

Here **S**_**i**_ is an *N* element vector, where the *j*-th element is the production rate of resource *i* in node *j* (which is fixed to a constant value of 0.5 in our model), **L** is the Laplacian matrix of the population network, *α*_**i**_ is an *N* element vector where the *j*-th element is the consumption rate of resource *i* in node *j* (which depends on the type of individual that occupies node *j*), and $c_{i}^{*}$ is an *N* element vector where the *j*-th element is the steady state concentration of resource *i* in node *j*. Therefore, at each time step, the steady state resource concentrations can by computed by solving a linear system of equations. Finally, the ecological fitnesses can be expressed as

$$\begin{array}{c} \tilde{r}=\alpha_{1}⊙c_{1}^{*}+\alpha_{2}⊙c_{2}^{*}, \end{array}$$ (18)

and the overall fitnesses as

$$\begin{array}{c} \tilde{f}=s⊙\tilde{r}, \end{array}$$ (19)

where ⊙ denotes element-wise multiplication and the *j*-th element of **s** gives the intrinsic fitness of the individual occupying node *j*. We then proceed to simulate population dynamics as described in the **Model** section.

**Fig 5:**
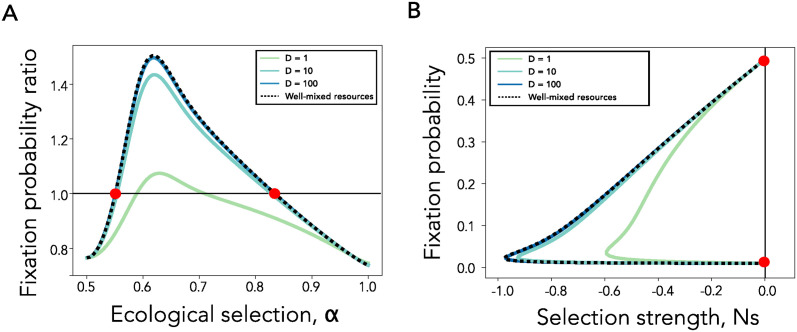
Robustness to diffusible resources. In this model, all resources are externally supplied at an equal rate and depleted at rates that vary spatially, determined by the mutational identity of the individual or subpopulation occupying that space. Resources are allowed to diffuse along the edges of the network with diffusion coefficient *D*. This coefficient is varied as in the legend. **Panel A.** Fixation probability ratios for a population with *N* = 100 and intrinsic selection coefficient *s* = −0.005. **Panel B.** Points of intersection between fixation probabilities between the network and well-mixed model for various diffusion coefficients, with the same parameters as in Panel A.

For a sufficiently large diffusion coefficient (*D* ≈ 100), the fixation probability ratio between a graph structure (*a*_*Bd*_ = 1.9) and a well-mixed population remains unchanged (blue solid line in Fig 5A, same analysis as in Fig 4A). The black dotted line shows the result from the earlier well-mixed resources model. An order of magnitude decrease in the diffusion coefficient (*D* ≈ 10) continues to have minimal effect on the fixation probability ratio (teal solid line in Fig 5A). However, when *D* ≈ 1, although the fixation probability is noticeably reduced compared to the well-mixed resources model, the key result wherein the ratio is greater than 1 for intermediate *α* holds (green solid line in Fig 5A). In Fig 5B, we repeat the analysis done in Fig 4B for the same amplifier spatial structure. We find that allowing for diffusible resources still allows the amplifier to increase the fixation probability of mutants having intrinsic deleterious selection up to *Ns* ≈ −1. For sufficiently small diffusion coefficient however, the limit of s that can be amplified is decreased to *Ns* ≈ −0.6. Finally, we also show that the analytic approximations for the fixation probabilities we derived earlier continue to hold when resources are allowed to diffuse (*D* = 10, Fig E in S1 Text).

### The role of tissue architecture in shaping rates of genetic hitchhiking: The example of Ductal Carcinoma

We next use our theoretical results and pancreatic ductal networks that have previously been inferred from imaging studies [36] and show that tissue architecture, in interplay with eco-evolutionary interactions, can have a strong influence on the dynamics of tumor cellular populations. For ductal carcinomas, where the branching topology of the ductal network dictates spatial constraints and mixing rates, tumor growth is highly spatially structured [20, 32]. We show that, on these structures, deleterious mutations that hitchhike with metabolic driver mutations can fix at higher rates, compared to other types of cancers having a different spatial context, for example well-mixed or lattice-structured tumor cellular populations.

**Fig 6:**
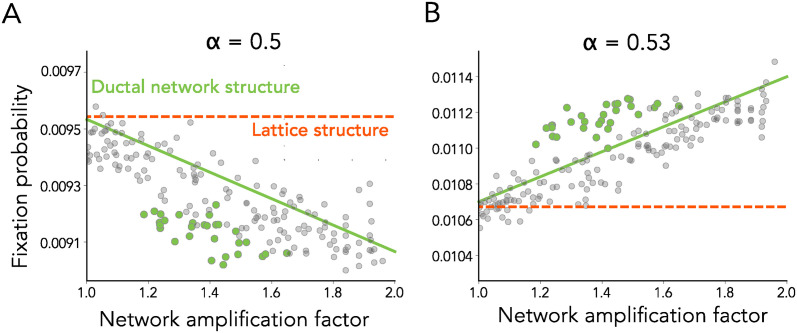
Probabilities of fixation on pancreatic ductal networks. The y-axis shows the probabilities of fixation of a deleterious mutant (*s* = −0.001) spreading on pancreatic ductal networks extracted from imaging data (green points), alongside other network families with similar amplification factors, such as Preferential Attachment and Bipartite graphs (grey points). We present two scenarios: the scenario of no ecological selection, where the deleterious mutant occurs on a genetic background with no differences in resource utilization between mutant and wild-type (**Panel A**) and the scenario of ecological selection, where the mutant also carries metabolic drivers, like mutations in the *KRAS* gene (**Panel B**). The red lines show the fixation probability of well-mixed / lattice graphs and the solid green lines represent the analytic approximation of the fixation probability (Eq (7)). For the cases of *α* = 0.5 and *α* = 0.53, the fixation probabilities shown by the points are estimated through simulation. Population size is assumed to be *N* = 100.

To this end, we use simulations together with our analytic approximations and compute probabilities of mutant fixation on these tree-like ductal networks. We assume mutants can either contain a metabolic driver which allows the cell to consume different resources (*α* > 0.5), or have no impact on a cell’s resource consumption (*α* = 0.5) and study the probability of fixation of deleterious intrinsic mutations hitchhiking on these two genetic backgrounds. In Fig 6A, we show probabilities of fixation of a mutant having intrinsic selection coefficient *s* = −0.001 and *α* = 0.5 and find that the pancreatic ductal tissue architecture leads to lower probabilities of mutant fixation (green line), compared to those in lattice-structured/well-mixed populations (red line). In contrast, in Fig 6B, we consider the fixation probability of the same deleterious intrinsic mutation, but now assume it occurs on the background of a metabolic driver, which can switch the cell’s metabolic profile and is under ecological selection, i.e. *α* = 0.53. We observe that, in contrast to the earlier case, the probability of fixation of these mutants is now higher on ductal topologies, compared to lattice-structured or well-mixed populations (which have the same probability of fixation), suggesting that tissue architecture can play a significant role in the frequencies of deleterious mutations accumulating in tumor cell populations.

### The time to fixation

**Fig 7:**
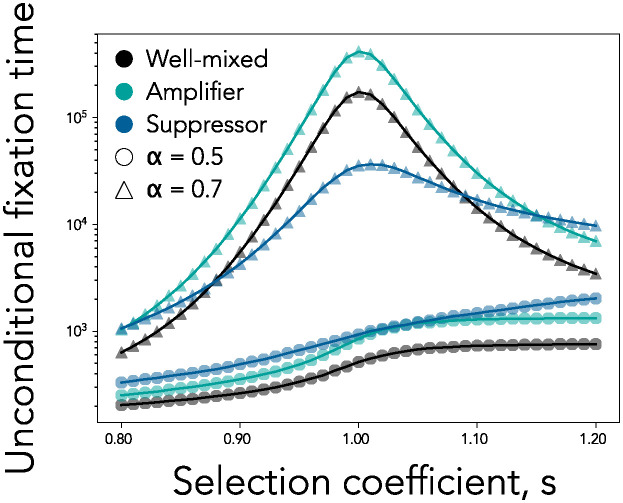
Well-mixed populations do not minimize fixation time. We plot the unconditional fixation time as a function of the intrinsic selection coefficient *s* for a well-mixed population, a suppressor, and an amplifier network. Here, *N* = 100. Each point of the graph shows the unconditional fixation time for a single network for a specific values of *s* and *α*, using 10^6^ simulation runs. Solid lines connect points corresponding to the same network. The shape of the point represents the value of ecological selection and the color represents the network type, as shown in the legend. In contrast to dynamics observed in the absence of ecological selection (*α* = 0.5), for *α* > 0.5, there exist network topologies that can decrease time at equilibrium and time to fixation, compared to well-mixed populations.

As the strength of ecological selection increases, the time to fixation of the new mutant also increases across all spatial structures. This is because the frequency-dependent selection increases the pull towards the unstable interior equilibrium. In the absence of ecological selection, it is known that the well-mixed populations minimize fixation time of new mutants in the population [30, 52]. Here however, we observe that, with ecological selection, it is possible for a population on a suppressor network to have a significantly shorter fixation time than the well-mixed population (Fig 7). There are two reasons for this. Firstly, when a mutant first appears in small quantities, in most cases it will immediately drift to extinction. However, the probability of immediate mutant loss is even larger on suppressor networks compared to well-mixed populations because suppressors decrease effective selection. Secondly, if the mutant successfully reaches equilibrium, the amount of ecological selection determines the strength of negative frequency-dependent selection pushing the ecotype frequencies towards the ecological equilibrium. Since suppressors decrease effective selection, the equilibrium is intrinsically less stable in these populations compared to well-mixed populations and the force of drift can more easily push the mutant towards the two absorbing equilibria.

## Discussion

We introduce a mathematical framework that combines evolutionary graph theory with a resource competition model to study how heterogeneous topological structure shapes evolutionary dynamics under global frequency-dependent ecological interactions. While the framework of graph-structured populations has been previously used in ecological models to model spatially heterogeneous resource distributions beyond simple structures of dispersal, prior models do not explicitly incorporate frequency-dependent ecological diversification pressures [54, 55]. We analyze the probability of fixation of a new ecotype mutant, characterized by two fitness axes: an intrinsic fitness axis and an ecological fitness axis, driven by the extent of niche separation. This model can be equivalently expressed as an evolutionary game on graphs in which payoff matrix elements are functions of the two parameters that control the strengths of ecological and intrinsic selection [28, 56]. A main difference between our model and previous literature on local frequency dependence dynamics on graphs is that here all resources are supplied by the environment and are not a diffusible public good produced through cooperation dynamics. Although some limited work exists in cases where replacement and frequency-dependent interactions occur over the entire network, both replacement and interaction networks are assumed to be sparse (low mean degree) [57]. This global frequency-dependence assumption also differentiates our model from others that assume each spatial niche hosts only one type of resource [54, 55].

This work builds on prior models of ecotype dynamics, each separately incorporating (and potentially examining in more detail) some of the integral parts of the model we build here. In some prior studies, all ecotype variants are assumed to be evolutionarily neutral [38, 53, 58] and/or analyze ecotype dynamics only in populations with highly symmetric, lattice-type spatial structures [53]. Separately, other previous models of consumer-resource dynamics on more complex graph structures assume a single predefined, spatially-heterogenous resource that all variants rely on, which effectively means they all occupy the same ecological niche [54, 55]. Other models allow for intrinsic fitness differences between different ecotype strains, however do not account for spatial structure [13].

While in most prior models that incorporate ecological niche differences, the focus is on understanding the population at equilibrium and factors such as the richness [59] and abundance distributions of species [60], an important distinction to our analysis is that we do not assume the limit of infinite population size and, in our model, genetic drift will eventually push the mutant to either fixation or loss from the population. This is similar to [13], where ecotype fixation is also possible and may occur if a sufficient number of intrinsic fitness-modifying mutations accumulate within a clade. Prior work has also only considered how network structure shapes either 1) constant selection dynamics in isolation or 2) frequency-dependent dynamics, without recognizing that even variants under frequency-dependent selection can carry an intrinsic constant fitness axis that spatial structure can act on.

Using the case without ecological differences as an explicit limit of our model, we show that the effects of an ecological, frequency-dependent fitness axis can affect the evolutionary properties of the network in non-intuitive ways. While amplifier spatial networks are known to suppress deleterious mutants [26, 29], here we show that weakly deleterious mutants, which can exploit ecological niche differences, can have increased fixation probabilities on amplifier networks, compared to well-mixed populations. We also show that the reverse is true for suppressor networks of transmission—the coupling of spatial topology and ecological selection can suppress weakly deleterious mutants. We show that the extent of this reversal in spatial structure effect is a non-monotonic function of niche overlap (i.e. strength of ecological selection) and we explain this result by considering the edge cases of weak and strong niche overlap.

In the regime of generalist populations, we can write out the effective fitness coefficient of the mutant as a function of the initial intrinsic selective coefficient, the ecological diversification strategy and the evolutionary topological properties of the network. The alternative edge case of little niche overlap (specialist ecotypes) corresponds to an evolutionary game on a graph under strong selection, which is generally not well understood. The fixation probability under arbitrary selection intensity is only known for some symmetric spatial structures [50, 61, 62]. Here we show that the net fixation probability can be decomposed into establishment and conditional fixation probabilities and that 1) establishment depends only on the strength of the frequency-dependent (ecological) selection and 2) conditional fixation depends only on the strength of the frequency-independent (intrinsic) selection. An analogous result is known for the fixation time under weak selection: 1) the conditional fixation time depends only on the frequency-dependent selection and 2) the unconditional fixation time depends only on frequency-independent selection [63]. Distinct establishment and conditional fixation subprocesses in specialist populations cause significant departures from evolutionary dynamics on graphs under effectively constant selection. For example, while the relationship between amplification and fixation probability is expected to be monotonic [29], we find that, for certain parameter regimes, it is graphs with intermediate amplification factor that can maximize the overall fixation probability of weakly deleterious mutants in specialist populations, because they achieve the best tradeoff between establishment and conditional fixation.

Beyond the theoretical interest of the questions presented here, we also use our theoretical results to show that tissue architecture can significantly shape eco-evolutionary dynamics of tumor cellular populations. For example, using a well-mixed model, [64] previously analyzed the variant allele frequency spectra of synonymous mutations from healthy blood and esophagus to quantify levels of positive selection and showed that, while in blood the majority of synonymous variants reaching high VAF do so by virtue of hitchhiking with a driver, clonal expansions in healthy esophagus residing elsewhere in the genome are rare. Exactly how the spatial structure of tissues shapes these observed differences and, more generally, tumor clonal dynamics, selective sweeps or rates of hitchhiking of deleterious mutations is an exciting under-explored area of research. While prior models of hitchhiking only consider driver mutations with a constant selective benefit [34, 35], or simple types of deme and stepping-stone-structured populations [65, 66], these models may be insufficient to understand hitchhiking for some types of cancers, since metabolic alterations are also commonly observed. For example, mutations in the *KRAS* gene occur often in ductal carcinoma [32, 33]. Here we show that the tree-like branching structure of pancreatic ductal carcinoma can reshape the spread and fixation of deleterious intrinsic fitness mutations on the background of metabolic drivers, compared to other types of tumors having a different spatial context.

For analytical conveniency and to curtail the number of parameters, our work has focused on a highly simplified model, which omits many of the complicating factors expected in either natural or laboratory settings. The optimal model choice depends on which parameters can be measured and future work could explore the effects of spatially-heterogenous resources [55, 67], the effects of cross-feeding [68, 69], higher-order multi-species interactions [70] and other more diverse modes of ecological interaction [71]. Our model also assumes that intrinsic fitness differences are directly linked to mutations that drive niche differentiation, rather than arising independently through secondary mutations. In addition, the strength of ecological selection is not allowed to evolve over time. These are all assumptions that need to be relaxed for a systematic understanding of eco-evolutionary dynamics. Our results provide an initial general framework for integrating ecological differences, population-genetic processes and complex patterns of transmission to study spatially heterogenous evolving ecosystems and lay the groundwork for engineering structures that select for communities of ecological, medical, or industrial utility.

## Materials and methods

Understanding which network properties shape eco-evolutionary dynamics is complicated by the fact that these properties are often correlated, hard to tune independently and differ across many network families. In previous work, we showed that linking network topology to evolutionary dynamics can be understood and analytically quantified by computing the network amplification factor, without having to keep track of all the lower-levels network properties [29, 30].

Here we use the approximation for the amplification factor from [29]. We can also compute the amplification factor empirically: using the Birth-death process, and starting with one single mutant with fitness (1 + *s*) invading a population of wild-type individuals with fitness 1, the amplification factor can be computed using the definition from [26] and solving

$$\begin{array}{c} \Phi =\frac{1-{\left(1+s\right)}^{-a_{Bd}}}{1-{\left(1+s\right)}^{-a_{Bd}N}} \end{array}$$ (20)

for *a*_*Bd*_, where Φ is the mutant’s fixation probability.

In this study, we use a combination of well-known complex network families using built-in generators from NetworkX [72] and also design graphs that allow us to tune the amplification factor and enable numerical tractability for our model, as detailed below.

### Preferential attachment graphs

For graphs with preferential attachment, nodes are added sequentially starting from a set *m* of initial nodes, until the population reaches size *N*. Each new node is added to the network and connected to other individuals with a probability proportional to the individual’s current degree to the power of a given parameter of preferential attachment, *β*. We use *m* ∈ {3, 5, 20}, and *β* ∈ [−3, 3].

### Preferential attachment stars

We introduce a family of symmetric preferential attachment-type graphs which allow exact computation of the mutant fixation probability, using numerical methods for absorbing Markov chains [48, 49]. These graphs strike a balance between exhibiting enough spatial heterogeneity, but also enough symmetry for analytical convenience. Specifically, a *N*-node PA star contains: 1) *i* core nodes, each connected to every other node in the graph, and 2) *N* − *i* leaf nodes, connected to all *i* core nodes. When *i* = 1, we recover the star graph, and when *i* = *N* − 1 we recover a well-mixed graph. Because only two variables are needed to represent any configuration of mutants on this type of graph, the mutant frequencies on core and leaf nodes, the symmetric structure of a PA star reduces the state space of the Markov process from 2^*N*^ states to (*i* + 1) ⋅ (*N* − *i* + 1) states, making numerical calculation possible. A PA star with N nodes and i core nodes is equivalent to a preferential attachment network with *m* = *i* and *β* = ∞.

### *k*-regular graphs

In a k-regular graph, every node has the same number of neighbors, *k*. We generate *k*-regular graphs using built-in generators from NetworkX, for different values of *k*, as presented in the text.

### Erdős Rényi random networks

The Erdős Rényi network is initialized with N disconnected nodes. Every pair of nodes is then connected independently with a probability *p*. We use NetworkX to generate these networks.

### Small world networks

The small world network is initialized with a regular ring lattice network of degree *k*. For every node, each of its neighbors are rewired with probability *p*. We use *k* ∈ {8, 12, 16}, and *p* ∈ [0, 1]. We generate small world networks using built-in generators from NetworkX.

### Bipartite graphs

Bipartite graphs have two sets of nodes *n*_1_ and *n*_2_ such that edges connect nodes across the two sets. We vary *n*_1_ from 1 to 50, and *n*_2_ = 100 − *n*_1_. We generate small world networks using built-in generators from NetworkX.

### Random geometric graphs

Random geometric networks are spatial networks with nodes sampled in a 2-dimensional space; we sample x coordinates for 50 nodes from N(0,0.25) and another 50 from N(3,0.25) and y-coordinates for all 100 nodes from N(0,0.25). We start with the lowest cut-off radius, resulting in a connected graph, and increase the cut-off radius until the complete graph is formed.

### Detour graphs

Detour graphs are initialized with a complete graph of size *n*_1_. Then one edge is replaced with a line graph of length *n*_2_. We use *n*_1_ ∈ [3, 99], and *n*_2_ = 100 − *n*_1_.

### Modified star graphs

The star graph consists of one center node connected to *N* − 1 outer nodes. We generate star graphs using built-in generators from NetworkX. Modified star graphs are initialized with a star graph of size N. Then *m* random edges are added to the graph. We use *m* = [50, 100, 200, 300].

## Supporting information

S1 Text File contains all the supplementary information for the article. (PDF)
