# Supplementary material for: Competition for resources can reshape the evolutionary properties of spatial structure
Source: PLoS Comput Biol. 2024 Nov 22;20(11):e1012542. doi: 10.1371/journal.pcbi.1012542 (PMC11623808; doi:10.1371/journal.pcbi.1012542)
Supplement: S1 Text — (PDF) [file pcbi.1012542.s001.pdf]

Supplementary Information

Competition for resources can reshape the evolutionary properties of spatial structure

Anush Devadhasan[1], Oren Kolodny[2], and Oana Carja *[1]

[1]Computational Biology Department, School of Computer Science, Carnegie Mellon University, Pittsburgh, PA, USA

[2]Department of Ecology, Evolution, and Behavior, E. Silberman Institute of Life Sciences, The Hebrew University of Jerusalem, Jerusalem, Israel

*To whom correspondence should be addressed. Email: oana@cmu.edu; oana.carja@gmail.com

SI Section A: Deriving the mutant relative fitness and frequency at equilibrium

From equation (1), the steady state concentrations of the two resources are

$$\begin{aligned} c_1^* &= \frac{S}{N(x\alpha + (1-x)(1-\alpha))} \\ c_2^* &= \frac{S}{N(x(1-\alpha) + (1-x)\alpha)}, \end{aligned} \tag{1}$$

where N is the population size and x is the mutant frequency in the population. Therefore, we can write the ecological mutant fitness as

$$\tilde{r_{mut}} = \alpha \cdot \mu(c_1^*(t)) + (1-\alpha) \cdot \mu(c_2^*(t)) = \frac{\alpha \cdot S}{N(x\alpha + (1-x)(1-\alpha))} + \frac{(1-\alpha) \cdot S}{N(x(1-\alpha) + (1-x)\alpha)} \tag{2}$$

and the wild-type fitness as

$$\tilde{r_{wt}} = (1-\alpha) \cdot \mu(c_1^*(t)) + \alpha \cdot \mu(c_2^*(t)) = \frac{(1-\alpha) \cdot S}{N(x\alpha + (1-x)(1-\alpha))} + \frac{\alpha \cdot S}{N(x(1-\alpha) + (1-x)\alpha)}, \tag{3}$$

where we assume $\mu(c_i^*(t)) = kc_i^*(t) = c_i^*(t)$. Therefore, the relative ecological fitness of the mutant is given by

$$r_{mut}(x) = \frac{\tilde{r_{mut}}}{\tilde{r_{wt}}} = \frac{\frac{\alpha}{x\alpha+(1-x)(1-\alpha)} + \frac{(1-\alpha)}{x(1-\alpha)+(1-x)\alpha}}{\frac{(1-\alpha)}{x\alpha+(1-x)(1-\alpha)} + \frac{\alpha}{x(1-\alpha)+(1-x)\alpha}}. \tag{4}$$

Note that multiplying the metabolic profiles by a constant value would leave (4) unchanged. To simplify the analysis, we show that relative mutant fitness can be written in a hyperbolic form as

$$r_{mut}(x) = a + \frac{b}{c+x}. \tag{5}$$

To determine the coefficients in (5), we use the following three conditions that $r_{mut}(x)$ must satisfy:

$$\begin{aligned} &r_{mut}(x=0.5) = 1, \\ &r_{mut}(x) = \frac{1}{r_{mut}(1-x)}, \\ &r_{mut}(x=0) = \frac{1}{2} \cdot \left(\frac{\alpha}{1-\alpha} + \frac{1-\alpha}{\alpha}\right), \end{aligned} \tag{6}$$

where the last condition follows from (4). We can write

$$r_{mut}(x=0.5) = a + \frac{b}{c+0.5} = 1 \Rightarrow a = \frac{1+2c-2b}{1+2c} \tag{7}$$

and plugging back into (5),

$$r_{mut}(x) = \frac{1+2c-2b}{1+2c} + \frac{b}{c+x}. \tag{8}$$

From the middle condition in (6), for $x = 1$, we get

$$\frac{1+2c-2b}{1+2c} + \frac{b}{c+1} = \frac{1}{\frac{1+2c-2b}{1+2c} + \frac{b}{c}}. \tag{9}$$

Solving for c and plugging back into (8), we find

$$c = \frac{b-1}{2} \Rightarrow r_{mut}(x) = \frac{b}{\frac{b-1}{2}+x} - 1. \tag{10}$$

Finally, using the last condition in (6), we can write

$$\frac{b}{\frac{b-1}{2}} - 1 = \frac{1}{2} \cdot \left(\frac{\alpha}{1-\alpha} + \frac{1-\alpha}{\alpha}\right) \Rightarrow b = (2\alpha-1)^{-2}. \tag{11}$$

Therefore,

$$r_{mut}(x) = \frac{(2\alpha-1)^{-2}}{\frac{1}{2}((2\alpha-1)^{-2}-1)+x} - 1. \tag{12}$$

Note that substituting α with $1-\alpha$ does not change (12). Therefore, while α can technically range from 0 to 1 in this model, it is sufficient to consider only the range 0.5 to 1 (or alternatively 0 to 0.5). The mutant relative fitness can alternatively be written as

$$r_{mut}(x) = \frac{(2\alpha-1)^{-2}+1-2x}{(2\alpha-1)^{-2}-1+2x}, \tag{13}$$

which more clearly establishes that this model is equivalent to an evolutionary game in which fitness is equal to payoff, with the payoff matrix

$$\begin{array}{c} \\ mut \\ wt \end{array} \begin{array}{c} \begin{array}{cc} \quad mut \quad & \quad wt \quad \end{array} \\ \begin{pmatrix} (2\alpha-1)^{-2}-1 & (2\alpha-1)^{-2}+1 \\ (2\alpha-1)^{-2}+1 & (2\alpha-1)^{-2}-1 \end{pmatrix} \end{array} .$$

The overall mutant relative fitness is then

$$f_{mut}(x) = (1+s) \cdot r_{mut}(x) = (1+s)\left(\frac{(2\alpha-1)^{-2}}{\frac{1}{2}((2\alpha-1)^{-2}-1)+x} - 1\right). \tag{14}$$

Note that at equilibrium, $f_{mut} = 1$. To find the equilibrium frequency x^*, we set $f_{mut} = 1$ and solve for x:

$$\begin{aligned} (1+s)\left(\frac{(2\alpha-1)^{-2}}{\frac{1}{2}((2\alpha-1)^{-2}-1)+x} - 1\right) &= 1 \\ \implies x^* = \frac{1}{2} + \frac{1}{2}(2\alpha-1)^{-2}\frac{s}{(2+s)}&. \end{aligned} \tag{15}$$

B: Deriving the fixation probability in the limit of the generalist population regime

In the limit of $\alpha \approx 0.5$, we can write

$$r_{mut}(x) = \frac{(2\alpha-1)^{-2}+1-2x}{(2\alpha-1)^{-2}-1+2x} \approx 1+2(2\alpha-1)^2(1-2x), \tag{16}$$

by Taylor expansion at $\alpha = 0.5$. The total relative fitness can then be approximated as

$$f_{mut}(x) = (1+s)\cdot(1+2(2\alpha-1)^2(1-2x)) \approx 1+s+2(2\alpha-1)^2(1-2x)+\mathcal{O}(s(2\alpha-1)^2), \tag{17}$$

where we can ignore terms $\mathcal{O}(s(2\alpha-1)^2)$, since intrinsic evolutionary selection is assumed to be weak ($|Ns| << 1$) throughout this paper. Similarly, the inverse eco-evolutionary fitness may be approximated as

$$f_{mut}^{-1}(x) \approx (1+s)^{-1}\cdot(1+2(2\alpha-1)^2(1-2x))^{-1} \approx 1-s-2(2\alpha-1)^2(1-2x)+\mathcal{O}(s(2\alpha-1)^2) \tag{18}$$

by Taylor expansion around $s=0$ and $\alpha = 0.5$.

The exact fixation probability in a well-mixed population is given by equation 1.16 in (1) as

$$P_{fix}^{WM} = \frac{1}{1+\sum_{j=1}^{N-1}\prod_{j=1}^{k}\frac{P_-(\frac{j}{N})}{P_+(\frac{j}{N})}} \tag{19}$$

where $P_-(\frac{j}{N})$ is the probability that the mutant frequency in the population decreases from $\frac{j}{N}$ to $\frac{j-1}{N}$ and the $P_+(\frac{j}{N})$ the probability that it increases from $\frac{j}{N}$ to $\frac{j+1}{N}$. Specifically, in order for the mutant frequency to increase by $\frac{1}{N}$ in 1 time step, a mutant must be sampled for birth proportional to fitness, and a wild-type must be randomly selected for death. We can write this probability as

$$P_+(\frac{j}{N}) = \frac{f_{mut}\big(\frac{j}{N}\big)}{w}\cdot\frac{j}{N}\cdot\big(1-\frac{j}{N}\big) \tag{20}$$

where w is the mean population fitness. Similarly, we can write

$$P_-(\frac{j}{N}) = \frac{1}{w}\cdot\frac{j}{N}\cdot\big(1-\frac{j}{N}\big), \tag{21}$$

and the ratio as

$$\frac{P_-(\frac{j}{N})}{P_+(\frac{j}{N})} = f_{mut}^{-1}(\frac{j}{N}). \tag{22}$$

Plugging back into P_{fix}^{WM}, we have

$$P_{fix}^{WM} = \frac{1}{1+\sum_{k=1}^{N-1}\prod_{j=1}^{k} f_{mut}^{-1}(x=j/N)} \approx \frac{1}{1+\sum_{k=1}^{N-1}\prod_{j=1}^{k}(1-(s+2(2\alpha-1)^2(1-\frac{2}{N}j)))}. \tag{23}$$

The product $\prod_{j=1}^{k} f^{-1}$ may be approximated as $1-\sum_{j=1}^{k}(s+2(2\alpha-1)^2(1-2x))$, by retaining only the $\mathcal{O}(s)$ and $\mathcal{O}((2\alpha-1)^2)$ terms in the product. This simplifies to

$$P_{fix}^{WM} \approx \frac{1}{N-(s+2(2\alpha-1)^2)\sum_{k=1}^{N-1}\sum_{j=1}^{k}1+\frac{4(2\alpha-1)^2}{N}\sum_{k=1}^{N-1}\sum_{j=1}^{k}j}. \tag{24}$$

The two double sums can be approximated as

$$\sum_{k=1}^{N-1}\sum_{j=1}^{k}1 = \frac{N(N-1)}{2} \approx \frac{N^2}{2} \tag{25}$$

and

$$\sum_{k=1}^{N-1}\sum_{j=1}^{k}j = \frac{1}{6}(N-1)N(N+1) \approx \frac{N^3}{6}, \tag{26}$$

by retaining only leading order terms in N, assuming large N. Using a Taylor expansion around $s=0$ and $\alpha=0.5$, we can write

$$P_{fix}^{WM} \approx \frac{1}{N-\frac{1}{2}sN^2-\frac{1}{3}(2\alpha-1)^2N^2} \approx \frac{1}{N}+\frac{1}{2}s+\frac{1}{3}(2\alpha-1)^2. \tag{27}$$

This approximation can be rewritten in terms of an effective selection coefficient as

$$P_{fix}^{WM} \approx \frac{1}{N}+\frac{1}{2}\left(s+\frac{2}{3}(2\alpha-1)^2\right) = \frac{1}{N}+\frac{1}{2}s_e, \tag{28}$$

since the constant weak selection fixation probability has the form $\frac{1}{N}+\frac{1}{2}s$ (see equation 7 in (2)). It is helpful to write the fixation probability in this form because we can then capture the effect of spatial structure using $s_e \to a_{Bd}\cdot s_e$.

C: Deriving approximations for $s^*(G)$ and P_{fix}^{G*} for large generalist populations

To find $s^*(G, \alpha)$, we first consider the difference in fixation probabilities and set it to 0

$$P_{fix}^{G} - P_{fix}^{WM} = (s + \frac{2}{3}(2\alpha - 1)^2)(a_{Bd} - 1) = 0. \quad (29)$$

For a network G, the only solution to this equation is

$$s = -\frac{2}{3}(2\alpha - 1)^2. \quad (30)$$

Therefore $s^*(G, \alpha) = s^*(\alpha) \approx -\frac{2}{3}(2\alpha - 1)^2$ for generalist populations. To get P_{fix}^{G*}, we plug $s^*(\alpha)$ into P_{fix}^{G} and can write

$$P_{fix}^{G*} \approx \frac{1}{N}. \quad (31)$$

In the limit of large population size, $P_{fix}^{G*} \to 0$.

D: Exact establishment and conditional fixation probabilities for specialist well-mixed and star-structured populations

The conditional fixation probability is the probability of mutant fixation starting from Nx^* mutants. In specialist populations, $x^* = 0.5$. The probability of mutant fixation given an initial mutant frequency of 0.5 can be computed using equation 1.20 in (1) as

$$P_{cfix}^{WM} = \frac{1 + \sum_{j=1}^{N/2-1} \prod_{k=1}^{j} (1+s)^{-1} \cdot r_{mut}^{-1}(x = k/N)}{1 + \sum_{j=1}^{N-1} \prod_{k=1}^{j} (1+s)^{-1} \cdot r_{mut}^{-1}(x = k/N)}. \tag{32}$$

The establishment probability is then given by

$$P_{est}^{WM} = \frac{P_{fix}^{WM}}{P_{cfix}^{WM}}, \tag{33}$$

which follows from equation 10 in the main text. The exact fixation probability on the star is given by equation 2.27 in (3) as

$$P_{fix}^{S} = \frac{1}{N} \cdot \frac{(N-1)(1-\beta(1)) + \gamma(0)}{1 + \sum_{j=1}^{N-2} (1-\gamma(j)) \prod_{k=1}^{j} \frac{\beta(k)}{\gamma(k)}}, \tag{34}$$

where

$$\gamma(i) \equiv \frac{(1+s) r_{mut}(x = i/N)}{N - 1 - i + (i+1)(1+s) r_{mut}(x = i/N)} \cdot \frac{N-1-i}{N-1} \tag{35}$$

and

$$\beta(i) \equiv \frac{1}{N - i + i(1+s) r_{mut}(x = i/N)} \cdot \frac{i}{N-1}. \tag{36}$$

On the star graph, there are 2 possible configurations of Nx^* mutants: 1) Nx^* mutants on the leaf nodes and a wild-type on the center node or 2) $Nx^* - 1$ mutants on the leaf nodes and a mutant on the center node. However, it is straightforward to see that configuration 1 can never be reached starting from a single mutant. Therefore the conditional fixation probability is equal to the probability of being absorbed in the state with N mutants starting from configuration 2. This is given by equation 2.23 in (3):

$$P_{cfix}^{S} = \frac{1 + \sum_{j=1}^{Nx^*-2} (1-\gamma(j)) \prod_{k=1}^{j} \frac{\beta(k)}{\gamma(k)}}{1 + \sum_{j=1}^{N-2} (1-\gamma(j)) \prod_{k=1}^{j} \frac{\beta(k)}{\gamma(k)}}. \tag{37}$$

From equation 10 in the main text, the exact establishment probability for a star can be computed by

$$P_{est}^{S} = \frac{P_{fix}^{S}}{P_{cfix}^{S}}. \tag{38}$$

E: Deriving an approximation for the establishment probability for well-mixed specialist populations

The establishment probability in a well-mixed specialist population simplifies to

$$P_{est}^{WM} = \frac{1}{1 + \sum_{j=1}^{N/2-1} \prod_{k=1}^{j} (1+s)^{-1} \cdot r_{mut}^{-1}(x = k/N)}, \tag{39}$$

by evaluating (33). We assume strong ecological selection: $r_{mut}^{-1}(x = 1/N) << 1$. Under this assumption, the sum of products is dominated by the leading order term, $(1+s)^{-1} r_{mut}^{-1}(x = 1/N)$. Under the assumption of weak intrinsic selection ($|Ns| << 1$), we can further write the leading order term as simply $r_{mut}^{-1}(x = 1/N)$. Then, the establishment probability may be approximated as

$$\begin{aligned} P_{est}^{WM} &= \frac{1}{1 + r_{mut}^{-1}(x = 1/N)} \\ &\approx 1 - r_{mut}^{-1}(x = 1/N). \end{aligned} \tag{40}$$

If we define the mutant invasion fitness as the fitness of the mutant when it has exactly one copy in the population, we can write

$$r_{inv}^{-1} \equiv r_{mut}^{-1}(x = 1/N). \tag{41}$$

More explicitly, we can write

$$\begin{aligned} P_{est}^{WM} &\approx 1 - \frac{(2\alpha - 1)^{-2} - 1 + \frac{2}{N}}{(2\alpha - 1)^{-2} + 1 - \frac{2}{N}} \\ &\approx \frac{2}{1 + (2\alpha - 1)^{-2}}, \end{aligned} \tag{42}$$

where in the last step we ignore $\mathcal{O}(N^{-1})$ terms.

F: Deriving an approximation for the conditional fixation probability for well-mixed specialist populations

Starting with the exact equation for the conditional fixation probability (32), we can write

$$P_{cfix}^{WM} = \frac{1 + \sum_{j=1}^{N/2-1} (1+s)^{-j} \prod_{k=1}^{j} r_{mut}^{-1}(x = k/N)}{1 + \sum_{j=1}^{N-1} (1+s)^{-j} \prod_{k=1}^{j} r_{mut}^{-1}(x = k/N)}, \tag{43}$$

where we use $\prod_{k=1}^{j}(1+s)^{-1} = (1+s)^{-j}$. The key observation necessary to simplify P_{cfix}^{WM} is that $\prod_{k=1}^{N-1} r_{mut}^{-1}(x = k/N) = 1$, which follows from the symmetry property of r_{mut}, i.e. $r_{mut}(x) = r_{mut}^{-1}(1-x)$. Therefore the last term in the sum in the denominator simplifies to $(1+s)^{-N+1} \prod_{k=1}^{N-1} r_{mut}^{-1}(x = k/N) = (1+s)^{-N+1}$. Pulling this term out of the sum, we can write

$$P_{cfix}^{WM} = \frac{1 + \sum_{j=1}^{N/2-1} (1+s)^{-j} \prod_{k=1}^{j} r_{mut}^{-1}(x = k/N)}{1 + (1+s)^{-N+1} + \sum_{j=1}^{N-2} (1+s)^{-j} \prod_{k=1}^{j} r_{mut}^{-1}(x = k/N)}. \tag{44}$$

If we assume strong ecological selection ($r_{mut}^{-1}(x = 1/N) = r_{inv}^{-1} << 1$), the $(1+s)^{-N+1}$ makes the largest contribution to the sum in the denominator. The next largest terms in the denominator and numerator are $\mathcal{O}(r_{inv}^{-1})$, so they may be ignored. This simplifies the problem to

$$\begin{aligned} P_{cfix}^{WM} &\approx \frac{1}{1 + (1+s)^{-N+1}} \\ &\approx \frac{1}{1 + (1+s)^{-N}}. \\ &\approx \frac{1 - (1+s)^{-N}}{1 - (1+s)^{-2N}}, \end{aligned} \tag{45}$$

where, in the last step, we multiply both numerator and denominator by $1 - (1+s)^{-N}$. We can alternatively write

$$P_{cfix}^{WM} \approx \frac{1}{2} + \frac{1}{4} Ns, \tag{46}$$

using Taylor expansion at $s = 0$.

G: Deriving approximations for $s^*(S,\alpha)$ and P_{fix}^{S*}, for large specialist populations

Using (40) and (46), we can approximate the total fixation probability in well-mixed specialist populations as

$$P_{fix}^{WM} \approx \left(1 - r_{mut}^{-1}(x=1/N)\right) \cdot \left(\frac{1}{2} + \frac{1}{4}Ns\right) \approx \frac{1}{2} + \frac{1}{4}Ns - \frac{1}{2}r_{mut}^{-1}(x=1/N) + \mathcal{O}(sr_{mut}^{-1}(x=1/N)), \quad (47)$$

where we ignore $\mathcal{O}(sr_{mut}^{-1}(x=1/N))$ terms. On the star graph, s is amplified as $s \rightarrow 2s$ (4) and $r_{mut}(x=1/N)$ as $r_{mut}(x=1/N) \rightarrow r_{mut}^2(x=1/N)$ (5), because s is weak and $r_{mut}(x=1/N)$ is strong. Therefore, the fixation probability on the star can be approximated as

$$P_{fix}^{S} \approx \frac{1}{2} + \frac{1}{2}Ns - \frac{1}{2}r_{mut}^{-2}(x=1/N). \quad (48)$$

To understand the point at which the fixation probabilities in the well-mixed and star populations intersect, we take the difference set to 0,

$$P_{fix}^{S} - P_{fix}^{WM} \approx \frac{1}{4}Ns + \frac{1}{2}\left(r_{mut}^{-1}(x=1/N) - r_{mut}^{-2}(x=1/N)\right) = 0 \quad (49)$$

and solve for Ns^*, which allows us to write

$$Ns^* \approx -2\left(r_{mut}^{-1}(x=1/N) - r_{mut}^{-2}(x=1/N)\right). \quad (50)$$

This equation showcases the value of Ns at which the fixation probabilities intersect for any given (strong) α. Since $r_{mut}(x=1/N)$ is large in the specialist regime, we can drop the $r_{mut}^{-2}(x=1/N)$ term and write

$$Ns^*(S,\alpha) \approx -2r_{mut}(x=1/N). \quad (51)$$

Plugging $r_{mut}^{-1}(x=1/N) \approx -\frac{1}{2}Ns^*(S,\alpha)$ into P_{fix}^{WM} gives us P_{fix}^{S*}

$$P_{fix}^{S*} \approx \frac{1}{2} + \frac{1}{2}Ns^*(S,\alpha). \quad (52)$$

References

[1] Traulsen A, Hauert C. Stochastic evolutionary game dynamics. Reviews of nonlinear dynamics and complexity. 2009 Jul 10;2:25-61.

[2] Sample C, Allen B. The limits of weak selection and large population size in evolutionary game theory. Journal of mathematical biology. 2017 Nov;75(5):1285-317.

[3] Hadjichrysanthou C, Broom M, Rychtár J. Evolutionary games on star graphs under various updating rules. Dynamic Games and Applications. 2011;1(3):386–407.

[4] Kuo YP, Nombela-Arrieta C, Carja O. A theory of evolutionary dynamics on any complex population structure reveals stem cell niche architecture as a spatial suppressor of selection. Nature Communications. 2024;15(1):4666.

[5] Lieberman E, Hauert C, Nowak MA. Evolutionary dynamics on graphs. Nature. 2005;433(7023):312–316.

Supplementary Material: Supplementary Figures

List of Figures

A **Robustness to asymmetric metabolic profiles and supply rates.** 16

B **The isothermal theorem holds under global frequency-dependent dynamics.** Each point shows the fixation probability of the mutant ecotype on a k-regular graph with mean degree as on the x-axis and N=100 for various values of α. Here $s = 0$. Fixation probabilities are computed using simulations with at least 2e6 replicates. 17

C **Approximations for establishment and conditional fixation probabilities in well-mixed and star-structured populations match exact calculations. Panel A** Dots show exact establishment probabilities on the complete and star graphs for varying ecological selection strength α and fixed intrinsic selection strength $s = 0.01$. Solid lines show the analytic approximations. Here $N = 100$. **Panel B** Dots show exact conditional fixation probabilities on the complete and star graphs for varying intrinsic selection strength s and fixed ecological selection strength $\alpha = 0.9$. Solid lines show the analytic approximations. Here $N = 100$. 18

D **Ecological interactions can reverse the role of amplifiers and suppressors for weakly deleterious mutants. The case of a suppressor topology.** The black lines are fixation probabilities estimated through 10^6 simulation runs for a suppressor network (amplification factor 0.47), for four different ecological strengths (values as depicted next to the orange dots). The grey lines shows the corresponding, exact fixation probabilities for a well-mixed population. The orange line follows the point of intersection between the well-mixed and suppressor populations, as ecological strength α is continuously varied. Population size $N = 50$. 19

E **Robustness of our results to diffusible resources.** In this model, all resources are externally supplied at an equal rate and depleted at rates that vary spatially, determined by the identity of the individual occupying that space. Resources are allowed to diffuse along the edges of the network with diffusion coefficient D=10. Dots show exact, numerically computed fixation probabilities in PA-star graphs with $N = 100$ and intrinsic selection coefficient $s = -0.005$ for $\alpha = 0.501$ in **Panel A.**, $\alpha = 0.65$ in **Panel B.**, and $\alpha = 0.99$ in **Panel C**. The solid black lines show the corresponding analytic approximations (equation (7) in **Panel A.** and **Panel B.** and equation (13) in **Panel C.**). 20

F **Residuals plot for the weak ecological selection approximation.** Residuals on the y-axis as a function of network amplification factor on the x-axis, across network families. Each dot represents the difference between the fixation probability of a single network, calculated using 10^6 simulation runs, and the analytic approximation given by equation (7). Here, $s = -0.001$, $N = 100$ and $\alpha = 0.53$. 21

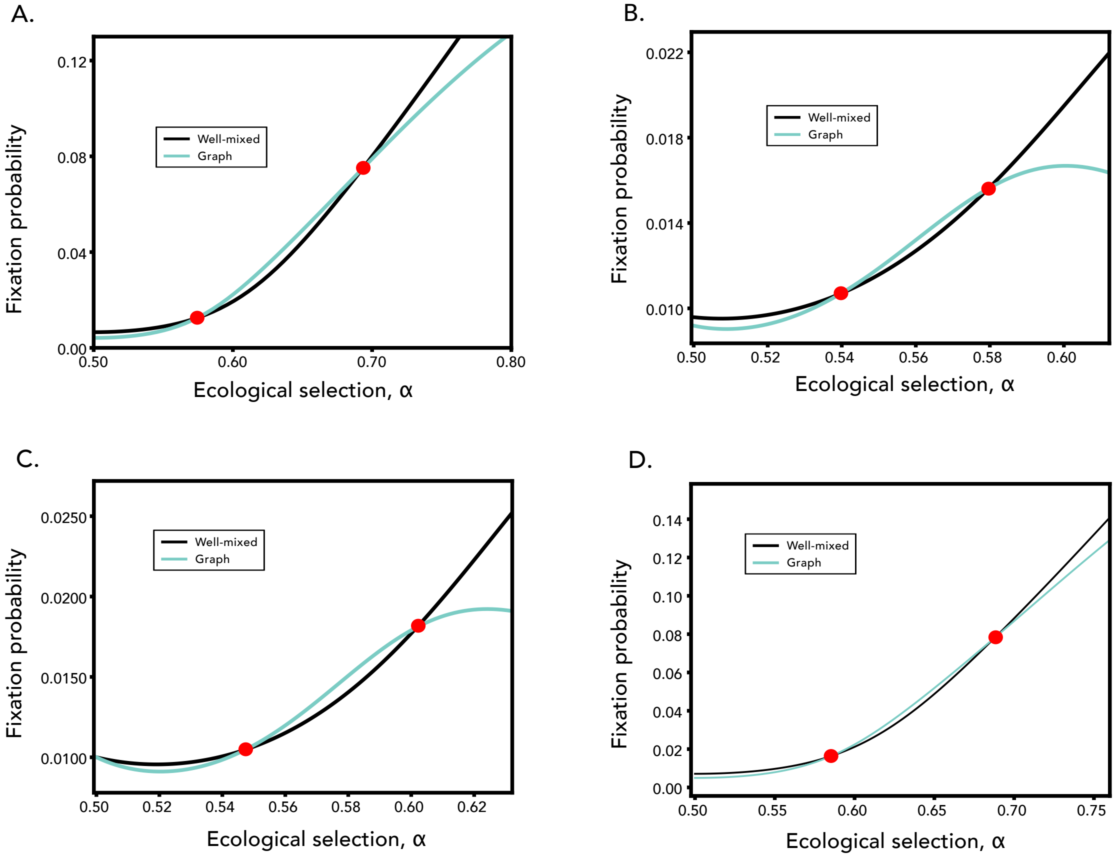

Supplementary Figure A: **Robustness to asymmetric metabolic profiles and supply rates. Panel A.** Fixation probabilities of a well-mixed (black) and star (green) population. Here, population size $N = 100$ and $s = -0.008$. **Panel B. Fixation probabilities under different metabolic profiles for each variant.** We assume the wild-type has metabolic profile $[\alpha, 1-\alpha]$ and the mutant $[1-(\alpha-\delta), \alpha-\delta]$. We set $\delta = -0.025$. The mutant has a intrinsic deleterious selection coefficient because it's metabolic profile is not as well matched to the supply rate as the wild-type. **Panel C. Fixation probabilities under different supply rates for each resource.** We assume that the mutant's preferred resource is supplied at rate $0.5+\delta$ and the other resource at $0.5-\delta$. We set $\delta = -0.025$. **Panel D. Fixation probabilities under deleterious intrinsic selection coefficient and structured resources.** Here, $N = 100$ and $s = -0.012$. All resources are externally supplied at an equal rate and depleted at rates that vary spatially, determined by the identity of individual occupying that space. Resources are allowed to diffuse along the edges of the network with diffusion coefficient $D = 2$.

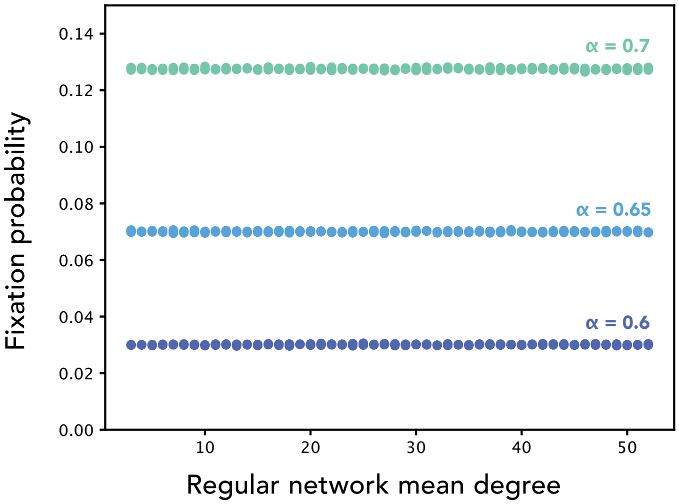

Supplementary Figure B: **The isothermal theorem holds under global frequency-dependent dynamics.** Each point shows the fixation probability of the mutant ecotype on a k-regular graph with mean degree as on the x-axis and N=100 for various values of α. Here $s = 0$. Fixation probabilities are computed using simulations with at least 2e6 replicates.

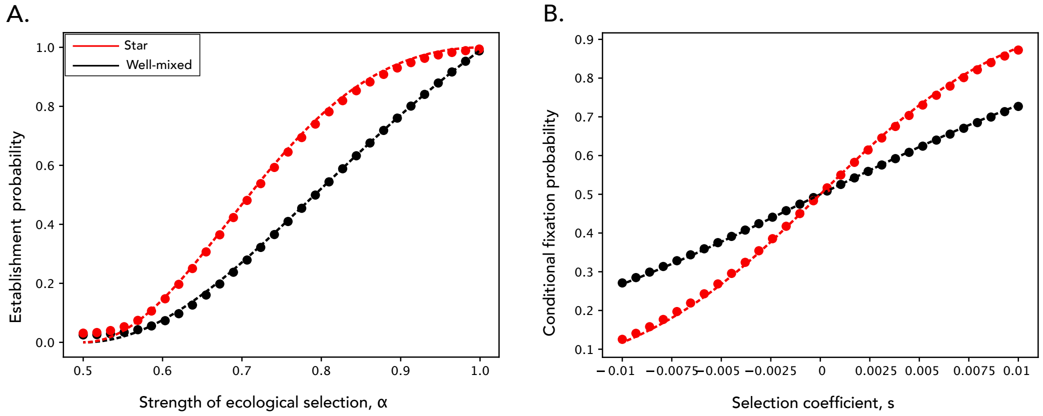

Supplementary Figure C: **Approximations for establishment and conditional fixation probabilities in well-mixed and star-structured populations match exact calculations. Panel A** Dots show exact establishment probabilities on the complete and star graphs for varying ecological selection strength α and fixed intrinsic selection strength $s = 0.01$. Solid lines show the analytic approximations. Here $N = 100$. **Panel B** Dots show exact conditional fixation probabilities on the complete and star graphs for varying intrinsic selection strength s and fixed ecological selection strength $\alpha = 0.9$. Solid lines show the analytic approximations. Here $N = 100$.

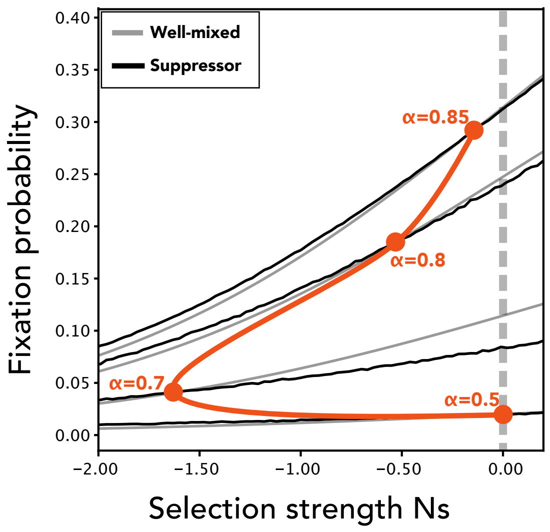

Supplementary Figure D: **Ecological interactions can reverse the role of amplifiers and suppressors for weakly deleterious mutants. The case of a suppressor topology.** The black lines are fixation probabilities estimated through 10^6 simulation runs for a suppressor network (amplification factor 0.47), for four different ecological strengths (values as depicted next to the orange dots). The grey lines shows the corresponding, exact fixation probabilities for a well-mixed population. The orange line follows the point of intersection between the well-mixed and suppressor populations, as ecological strength α is continuously varied. Population size $N = 50$.

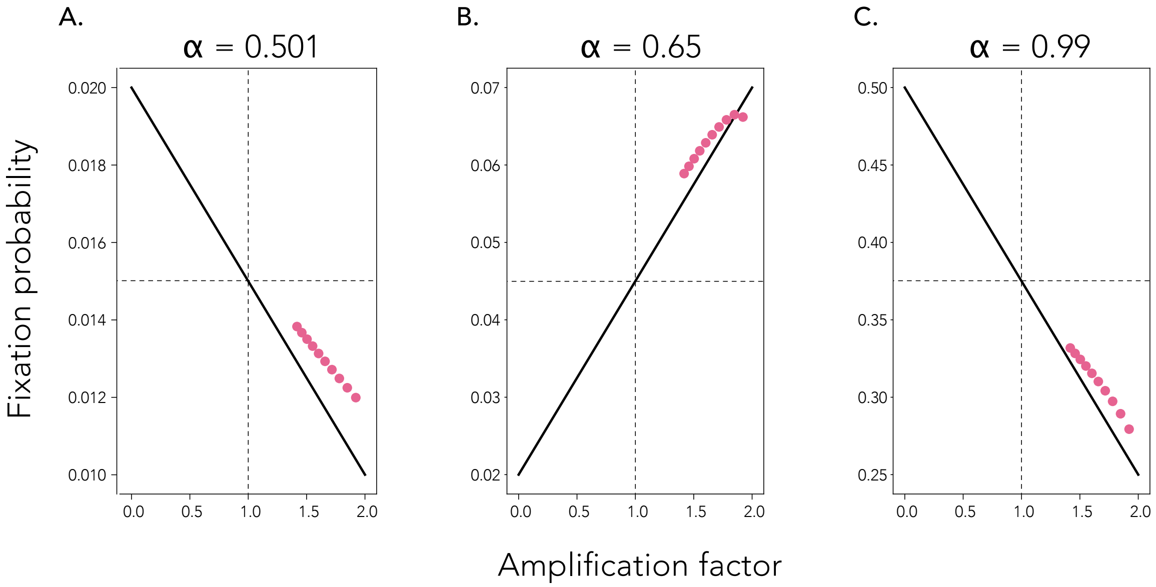

Supplementary Figure E: **Robustness of our results to diffusible resources.** In this model, all resources are externally supplied at an equal rate and depleted at rates that vary spatially, determined by the identity of the individual occupying that space. Resources are allowed to diffuse along the edges of the network with diffusion coefficient D=10. Dots show exact, numerically computed fixation probabilities in PA-star graphs with $N = 100$ and intrinsic selection coefficient $s = -0.005$ for $\alpha = 0.501$ in **Panel A.**, $\alpha = 0.65$ in **Panel B.**, and $\alpha = 0.99$ in **Panel C**. The solid black lines show the corresponding analytic approximations (equation (7) in **Panel A.** and **Panel B.** and equation (13) in **Panel C.**).

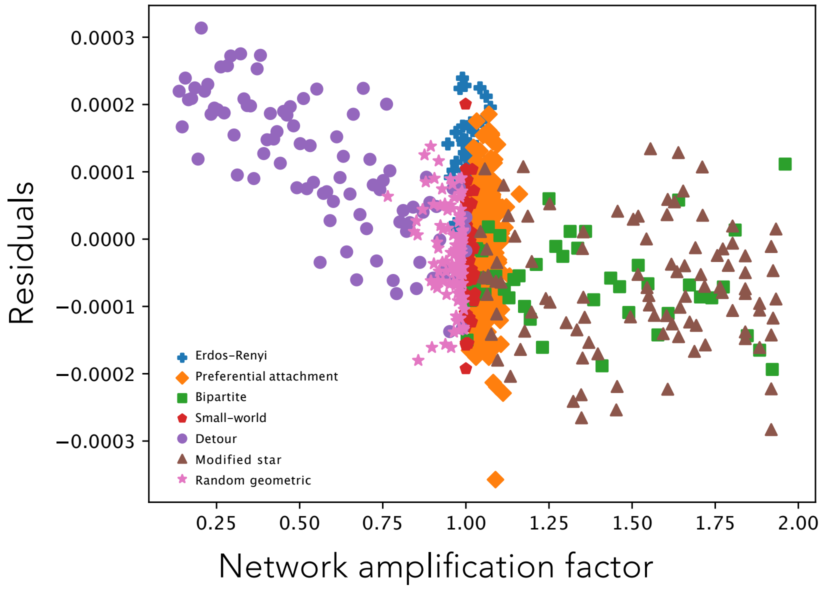

Supplementary Figure F: **Residuals plot for the weak ecological selection approximation.** Residuals on the y-axis as a function of network amplification factor on the x-axis, across network families. Each dot represents the difference between the fixation probability of a single network, calculated using 10^6 simulation runs, and the analytic approximation given by equation (7). Here, $s = -0.001$, $N = 100$ and $\alpha = 0.53$.
